# Comparative phytochemical, antioxidant, and hemostatic studies of leaf and stem extracts of *Rhazya stricta* Decne. in human plasma and human peripheral blood mononuclear cells *in vitro*

**DOI:** 10.3389/fmolb.2026.1786802

**Published:** 2026-05-11

**Authors:** Arafa I. Hamed, Amal A. A. Mohamed, Mohamed Ali Ben Aissa, Bogdan Kontek, Magdalena Kluska, Katarzyna Woźniak, Mariusz Kowalczyk, Iwona Kowalska, Wiesław Oleszek, Beata Olas

**Affiliations:** 1 Phytochemistry Laboratory, Department of Botany, Faculty of Science, Aswan University, Aswan, Egypt; 2 Department of Chemistry, College of Science, Qassim University, Buraydah, Saudi Arabia; 3 Department of General Biochemistry, Faculty of Biology and Environmental Protection, University of Lodz, Łódź, Poland; 4 Department of Molecular Genetics, Faculty of Biology and Environmental Protection, University of Lodz, Łódź, Poland; 5 Department of Phytochemistry, Institute of Soil Science and Plant Cultivation, State Research Institute, Puławy, Poland; 6 Centre for Medicinal Plant Cultivation, Institute of Soil Science and Plant Cultivation, State Research Institute, Puławy, Poland

**Keywords:** alkaloids, anticoagulant activity, hemostasis, oxidative stress, rhayza stricta, ultra-performance liquid chromatography–electrospray ionization–MS/MS

## Abstract

**Introduction:**

*Rhazya stricta* Decne. is considered an important medicinal plant that is rich in secondary metabolites containing anticancer alkaloids. Several indole alkaloid classes have been identified from the various parts of *R. stricta*, but the cytotoxic potentialities of only a few of these metabolites are known.

**Methods:**

In this study, an applied analytical method was used to determine the alkaloids in *R. stricta* from the stem (RS) and leaf (RL) and their extracts.

**Results and Discussion:**

This study tentatively elucidated 10 new compounds of indole alkaloids from the various parts of *R. stricta*. Among them, six alkaloidal glycosides had not been detected in natural resources. This investigation also examined the *in vitro* protective results of the four *R. stricta* stem (A–D) and leaf (A′–D′) extracts, each having a different group of compounds—indole alkaloids, against oxidative stress activated by H_2_O_2_/Fe^2+^ in human plasma *in vitro*. In addition, we estimated the effect of these plant extracts on DNA fragmentation in human peripheral blood mononuclear cells (PBMCs). Another aim of these *in vitro* experiments was to determine the result of A–D and A′–D′ on selected hemostatic parameters of human plasma, such as the activated partial thromboplastin time, prothrombin time, and thrombin time, and on the viability of PBMCs. Based on our results, we demonstrate for the first time that tested extracts of the leaves and stems of *R. stricta* containing different indole alkaloid compounds (especially two tested preparations from *R. stricta* leaf—C′ and D′) are good antioxidant *in vitro* models, depending on the dosage, and they may have some promising actions *in vivo.* For example, we observed a significant difference in the level of DNA damage induced by H_2_O_2_ in the experiment with A–D and A′–D′. Two tested extracts from *R. stricta* leaf (C′ and D′, at all concentrations used) were also found to protect plasma against H_2_O_2_/Fe^2+^—induced lipid peroxidation. In addition, preparation C′ does not induce cytotoxicity. The potential of the metabolomics of extract C′ may be correlated to the presence of rhazisidine, secamine, and their derivatives.

## 1 Introduction

Alkaloids are a wide range of naturally occurring metabolites that occur in medicinal plants that have one or more nitrogen atoms in their structures. Alkaloids naturally present in foods (e.g., certain indole alkaloids in cruciferous vegetables and coffee) may exert mild stimulatory or antioxidant effects, depending on their concentration and bioavailability (Liu et al., 2007; Baeshen et al., 2009; Baeshen et al., 2010; Ahmad et al., 1970; Ahmad et al., 1971; Ahmad et al., 1979; Ahmad et al., 1983; Ahmed et al., 2015; Ahmed et al., 2018; Andersen et al., 1987; Atta-ur-Rahman, 1984; Atta-ur-Rahman et al., 1987; Rehman and Rahman, 1996; Rahman et al., 1989; Marwat et al., 2012; Obaid et al., 2017; Abdul-Hameed et al., 2022; Pravin et al., 2025; Aljawdah et al., 2025; Baeshen et al., 2022; Adamski et al., 2020; Rajashekar, 2023).

Indole alkaloids are five-membered rings with one hetero-nitrogen atom and are one of the principal classes of alkaloid. Indole alkaloids are typically found in the families Rubiaceae and Apocynaceae (Liu et al., 2007). They exhibit various pharmacological activities, including antihypertensive, antimicrobial, and antitumor properties (Baeshen et al., 2009; Pravin et al., 2025; Aljawdah et al., 2025; Baeshen et al., 2022). High levels of indole alkaloids (e.g., harman and norharman) can exhibit neurotoxic effects, particularly in sensitive individuals (Liu et al., 2007; Baeshen et al., 2009; Baeshen et al., 2010; Ahmad et al., 1970; Ahmad et al., 1971; Ahmad et al., 1979; Ahmad et al., 1983; Ahmed et al., 2015; Ahmed et al., 2018; Andersen et al., 1987; Atta-ur-Rahman, 1984; Atta-ur-Rahman et al., 1987; Rehman and Rahman, 1996; Rahman et al., 1989; Marwat et al., 2012; Obaid et al., 2017; Abdul-Hameed et al., 2022; Adamski et al., 2020; Rajashekar, 2023).

The genus *Rhazya* comprises two species (Migahid, 1989; Mandaville, 1990; Chaudhary and Al-Jowaid, 1999; Bukhari et al., 2017; Ali et al., 2000): *R. stricta* Decne (syn. *R. greissii* Decne) and *R. orientalis* Decne (syn. *Amsonia orientalis* Decne). *R. stricta*, commonly known as “Harmal”, is a parched plant that belongs to the family Apocynaceae. The *Rhazya* genus is named after a Muslim scientist, Abu Bakr Mohammed bin Zakariya Al-Razi, known in Europe under the Latinized name of *Rhazes*. The plant is an erect shrub with glabrous leaves, yellow-green and broadly linear-lanceolate, which wrinkle after drying. It has a smooth central stem and dense semi-erect branches; the leaves alternate; its flowers are white in short branched cymes; its fruit has pale yellow follicles; and its seeds are short-winged (Migahid, 1989; Mandaville, 1990; Chaudhary and Al-Jowaid, 1999; Bukhari et al., 2017; Ali et al., 2000).

The medicinal plant *R*. *stricta* is known to be a rich natural source of indole alkaloids, monoterpenoid indole alkaloids, triterpenes, phenols, and glycosides (Baeshen et al., 2009; Baeshen et al., 2010; Ahmad et al., 1970; Ahmad et al., 1971; Ahmad et al., 1979; Ahmad et al., 1983; Ahmed et al., 2015; Ahmed et al., 2018; Andersen et al., 1987; Atta-ur-Rahman, 1984; Atta-ur-Rahman et al., 1987; Rehman and Rahman, 1996; Rahman et al., 1989; Marwat et al., 2012; Obaid et al., 2017; Abdul-Hameed et al., 2022). Long ago, *R. stricta* was used in traditional medicine to treat chronic rheumatism and fever (Rahman et al., 1989). In previous studies, *R. stricta* extracts and the pure alkaloidal metabolites displayed positive anti-diabetic effects (Baeshen et al., 2010; Ahmed et al., 2015; Ali et al., 2000; Ali, 1997) and antioxidant and anticancer properties (Baeshen et al., 2012; Mukhopadhyay et al., 1981; Gu and Zakarian, 2010; El-Awady et al., 2015; Shahat et al., 2016; Al-Dabbagh et al., 2018; Shaer, 2019). Other biological properties of various parts of *R. stricta* are less recognized, especially toward the cardiovascular system. Indole alkaloids are commonly analyzed using HPLC- or UPLC-based analytical methods, and UPLC–Q-TOF mass spectrometry achieves rapid and sensitive identification of indole alkaloids (Schnoes et al., 1962; Li et al., 2011; Nordström et al., 2006; Esquenazi et al., 2009; Demarque et al., 2016; Hamed et al., 2022; Ben Said et al., 2023; Mahida et al., 2026; Fu et al., 2025; Youssif et al., 2024; Akhgari et al., 2015a; Akhgari et al., 2015b; Baeshen et al., 2014; Baeshen et al., 2015; Baeshen et al., 2023).

Oxidative stress is a key factor in the progression of a range of human illnesses, such as cancer and cardiovascular diseases. Phytochemicals found in various plants have demonstrated significant potential in the treatment and prevention of diseases associated with oxidative stress (Milev et al., 2025; Al-Naqeb et al., 2024; Maliar et al., 2023; Diogo Gonçalves et al., 2025; Ladeira et al., 2024). So far, there are no data on the effect of *R. stricta* indole alkaloids on the physiology of various elements of human blood, including their interaction with human plasma and the hemostatic system. Therefore, for the first time, our study aimed to illustrate the *in vitro* protective results of the four *R. stricta* stem (A–D) and leaf (A′–D′) extracts, each containing a different structural secondary metabolite, against oxidative stress activated by H_2_O_2_/Fe^2+^ (donor of hydroxyl radicals) in human plasma. The Fenton reaction, driven by the interaction of Fe^2+^ with H_2_O_2_, generates hydroxyl radicals (•OH), which constitute one of the most potent oxidizing species in biological systems. In the initial step, Fe^2+^ is rapidly oxidized by H_2_O_2_, producing Fe^3+^, hydroxide ion, and the highly reactive •OH radical. This step is central to the reaction’s oxidative capacity as •OH readily attacks lipids, proteins, and nucleic acids. Ferric iron formed in this process is subsequently reduced back to Fe^2+^ by a second molecule of H_2_O_2_, yielding hydroperoxyl radicals (•OOH) and allowing the continuous cycling of iron in the Fe^2+^/Fe^3+^ redox couple. Overall, the net conversion of H_2_O_2_ results in the simultaneous formation of •OH and •OOH radicals. The reaction proceeds most efficiently under acidic conditions, where Fe^2+^ remains soluble and available for redox cycling. At higher pH, iron precipitation inhibits radical formation. Given the extreme oxidizing power of •OH, which surpasses most other biologically relevant oxidants, the Fenton system represents a major source of oxidative stress in both chemical assays and cellular environments (Maliar et al., 2023).

We investigated three different parameters of oxidation in human plasma: lipid peroxidation (detected by thiobarbituric acid reactive substances—TBARSs), protein carbonylation, and thiol group level. In addition, we demonstrated the effect of these plant extracts on DNA damage (by applying the comet assay) in PBMCs. Another goal of our experiments conducted *in vitro* was to determine the effect of the A–D and A′–D′ extracts on selected hemostatic parameters of human plasma (the stimulated partial thromboplastin time (APTT), prothrombin time (PT), and thrombin time (TT)) and on the viability of PBMCs.

The goal of this study is to describe a rapid, sensitive ultra-performance liquid chromatography–electrospray ionization–quadrupole time-of-flight (UPLC–ESI–Q-TOF) technique for detecting and tentatively identifying alkaloid metabolites from the leaves and stems of *R. stricta*, which represent an important traditional natural resource for medicinal drugs.

## 2 Materials and methods

### 2.1 Chemicals

Dimethylsulfoxide (DMSO), thiobarbituric acid (TBA), 4′,6-diamidino-2-phenylindole (DAPI), low-melting-point (LMP) and normal-melting-point (NMP) agarose, phosphate-buffered saline (PBS), and H_2_O_2_ were purchased from Sigma-Aldrich (St. Louis, MO, United States). All other reagents represented analytical grade and were provided by commercial suppliers.

### 2.2 Plant material

The plant materials were collected from Qassim Province in March 2019 at 25.8700° N, 43.5001° E in Saudi Arabia. The plant material was identified by Prof. Arafa Hamed according to Täckholm (1974) and was compared with voucher #NATKSU-108 (Department of Botany and Microbiology, King Saud University, Saudi Arabia). Our voucher plant (#13) was deposited in the Chemistry Department, College of Science, Qassim University, Al Rass, Kingdom of Saudi Arabia. The different parts of the collected materials (roots, stems, and leaves) were then separated to complete the drying process at room temperature, far from direct sunlight.

#### 2.2.1 Plant description

The genus *Rhazya* comprises two species (Ali et al., 2000): *R. stricta* Decne (syn. *Rhazya greissii* Decne) and *R. orientalis* Decne (syn. Amsonia *orientalis* Decne). *R. stricta*, commonly known as “Harmal”, is a parched plant that belongs to the family Apocynaceae. The *Rhazya* genus is named after a Muslim scientist, Abu Bakr Mohammed bin Zakariya Al-Razi, known in Europe under the Latinized name of *Rhazes*. The plant is an erect shrub with glabrous leaves, yellow-green and broadly linear-lanceolate, which wrinkle after drying. It has a smooth central stem and dense semi-erect branches; the leaves alternate; its flowers are white in short branched cymes; its fruit has pale yellow follicles; and its seeds are short-winged (B).

### 2.3 Preparation of extracts A–D and A′–D′

The leaves and stems were dried for 2 weeks at room temperature (35 °C), avoiding direct sunlight. The dried leaves of *R. stricta* (RL-250 g), after grinding, were exhaustively extracted four times using 80% MeOH (MeOH: H_2_O, 80:20) by maceration at room temperature (24 h, 35 °C). The crude extract thus obtained was concentrated under reduced pressure at 600 C using a Witeg Vertical Rotary Evaporator 20–280 rpm, 500 mL, to obtain a syrupy consistency (65 g, 22.4% from the dried leaves). Part of the crude extract (10 g) was dissolved in a distilled water liquor and loaded onto a preconditioned short C18 column (6 × 10 cm, 60 lm C18, Backer) and eluted with H_2_O (100%, washing), 20% MeOH (MeOH: H_2_O, 20:80, **A, 1.7 g**), 40% MeOH (MeOH: H_2_O, 40:60, **B, 2.5 g**), 60% MeOH (MeOH: H_2_O, 60:40, **D**, **3.6 g**), 80% MeOH (MeOH: H_2_O, 80:20, **C**, **1.5 g**), and 100% MeOH (washing). The same procedures were applied for the dried stem parts (**RS**-250 g), which yield 77 g (30.8% from the dried stems) after removing the used solvent. Part of the RS crude extract (10 g) was fractionated in the same manner and yielded the extracts **A**’ (20% MeOH**, 1.2 g**), **B’** (40% MeOH, **2.7 g)**, **C’** (60% MeOH, **3.7 g**), **D’** (80% MeOH, **1.6 g**), and 100% MeOH (washing). All extracts (**A**–**D** and **A′** –**D)** were concentrated using the same method and investigated by C-18 TLC using the solvent system CH_3_CN:H_2_O (30:70 and 40:60) and sprayed with Dragendorff’s reagent, which is used for visualizing alkaloids. **A**–**D** and **A′**–**D′** tested positive for alkaloid.

### 2.4 High-resolution LC-MS analyses of plant extracts

High-resolution LC-MS analyses of plant extracts were achieved according to our previous methods (41, 42, supplementary data (Liu et al., 2007)).

### 2.5 Preparation of stock solutions of plant extracts for bioassay

Stock solutions of *R. stricta* extracts A–D and A′–D′ were prepared with 50% (v/v) aq. DMSO, a universal solvent for different plant metabolomics. The final DMSO concentration in the tested samples was below 0.05% (v/v). To achieve 50% DMSO in the tested samples, 0.05% (v/v) was diluted 1,000 times during the tests.

### 2.6 Isolation of human plasma

Plasma was obtained from a blood bank in Łódź (Poland), and it came from regular, medication-free donors. The donors did not drink alcohol or take medicine (including antiplatelet drugs, aspirin and its derivatives, or anticoagulants) for 2 weeks before blood collection. The Bioethics Committee at the University of Łódź approved the protocol for research on human subjects (number 2/KBBN-UŁ/II/2016). The research was also conducted according to the guidelines of the Helsinki Declaration for Human Research, with the approval of the committee.

Each sample (plasma) taken for analysis came from a different donor and was an independent trial. To measure the parameters of hemostasis and auto-oxidation of biomolecules (lipids and proteins), the plasma was incubated at 37 °C for 30 min with the tested plant extracts (concentration range 0.5–50 μg/mL). To measure the oxidative stress parameters, the plasma was pre-incubated at 37 °C for 5 min with the tested plant extracts (concentration range 0.5–50 μg/mL) and then treated with a final concentration of 4.7 mM H_2_O_2_/3.8 mM Fe_2_SO_4_/2.5 mM EDTA (25 min, at 37 °C). “Control negative” refers to plasma not treated with H_2_O_2_/Fe^2+^; “control positive” refers to plasma treated with H_2_O_2_/Fe^2+^.

The protein concentration was calculated by measuring the absorbance of the tested samples at 280 nm, according to Whitaker and Granum (1980) and using the assay of Bradford (1996).

### 2.7 PBMCs isolation

PBMCs were isolated from leucocyte buffy coats obtained from healthy, non-smoking donors provided by the Blood Bank in Łódź, Poland, as described by Kluska et al. (2019). The leucocyte-buffy coat was diluted in a 1:1 ratio in PBS and centrifuged in a density gradient of Lymphosep (Cytogen, Zgierz, Poland) at 200 × g for 20 min at room temperature. PBMCs were then collected and washed three times by centrifugation in PBS. The pellet of the cells was resuspended in RPMI 1640 medium (Lonza, Basel, Switzerland).

All procedures were approved by the Research Ethics Committee of the University of Łódź (approval no. 12/KEBN-UŁ/I/2024–2025).

### 2.8 Markers of oxidative stress

#### 2.8.1 Lipid peroxidation measurement

Lipid peroxidation was quantified by measuring TBARS concentration as per Wachowicz (1984) and Bartosz (2013). After incubation, the samples were mixed with an equal volume of cold 15% (v/v) trichloroacetic acid in 0.25 M HCl and 0.37% (v/v) TBA in 0.25 M HCl and then immersed in a boiling water bath for 15 min. After cooling, the absorbance was measured at 535 nm using the SPECTROstar Nano Microplate Reader (BMG LABTECH, Germany). The TBARS concentration was calculated using the molar extinction coefficient (ε = 156,000 M^-1^ cm^-1^) and was expressed as nmol/mL of plasma.

#### 2.8.2 Carbonyl group measurement

The carbonyl groups were determined in plasma protein according to Levine et al. (1990) and Bartosz (2013). The absorbance measurement (at 375 nm) was performed using a SPECTROstar Nano Microplate Reader (BMG LABTECH). The carbonyl group concentration was calculated using a molar extinction coefficient (ε = 22,000 M^-1^ cm^-1^) and was expressed as nmol/mg of plasma protein.

#### 2.8.3 Thiol group measurement

After incubation, the test samples were transferred to a 96-well plate at 20 μL, followed by the addition of 20 μL of sodium dodecyl sulfate (SDS), and mixed thoroughly. Successively, 160 μL of 10 mM phosphate buffer (pH 8.0) was added to all samples and mixed thoroughly. Absorbance was measured at a wavelength λ = 412 nm (A_0_), and 16.6 μL Ellman’s reagent (5,5′-dithio-bis-(2-nitrobenzoic acid); DTNB) was added. The 96-well plate was incubated for 60 min (temperature 37 °C). After incubation, absorbance was measured at a wavelength λ = 412 nm (A_1_) using the SPECTROstar Nano Microplate Reader (BMG LABTECH) as per Ando and Steiner (1973), Ando et al. (1973), and Bartosz (2013). The absorbance difference A_1_-A_0_ was calculated. The thiol group concentration was calculated using a molar extinction coefficient (ε = 13 600 M^-1^ cm^-1^) and was expressed as nmol/mg of plasma protein.

#### 2.8.4 DNA oxidative damage analysis

DNA oxidative damage was assessed using the alkaline comet assay, following the method described by Singh et al. (1988) as adapted by Kluska et al. (2019) and Tokarz et al. (2016). In brief, PBMCs were adjusted to a concentration of 1 × 10^5^ cells/mL and incubated with *R. stricta* extracts for 2 h at 37 °C. The cells were then treated with 20 µM H_2_O_2_ for 15 min on ice.

Following treatment, the cells were centrifuged, resuspended in 0.75% LMP agarose, and spread onto microscope slides pre-coated with 0.5% NMP agarose. The slides were subsequently immersed in lysis solution (2.5 M NaCl, 0.1 M EDTA, 10 mM Tris, and 1% Triton X-100; pH 10) for 1 h. DNA unwinding was performed in ice-cold alkaline buffer (300 mM NaOH, 1 mM EDTA; pH > 13) for 20 min, followed by electrophoresis in ice-cold buffer of 30 mM NaOH and 1 mM EDTA for 20 min at 0.73 V/cm (29 mA).

After electrophoresis, the slides were rinsed, stained with DAPI (2 μg/mL), and examined using a fluorescence microscope. Fluorescent imaging was conducted at ×200 magnification using an Eclipse fluorescence microscope (Nikon, Tokyo, Japan) equipped with a ProgRes MF cool monochrome camera (JENOPTIK, Jena, Germany) and connected to a Lucia Comet Assay 7.30 image analysis system (Laboratory Imaging, Prague, Czech Republic). For each sample, 50 comets were randomly selected for analysis, and the percentage of DNA in the tail comet was quantified as an indicator of DNA damage. Two independent experiments were performed.

### 2.9 Cell viability

Cell metabolic activity was evaluated using the resazurin reduction assay as per O’Brien et al. (2000). PBMCs were plated in 96-well culture plates at a density of 5 × 10^4^ cells per well and exposed to *R. stricta* extracts at final concentrations of 0.5, 5, and 50 μg/mL for 24 h under standard culture conditions (37 °C, 5% CO_2_). After treatment, 10 µL of resazurin solution (2 mg/10 mL in PBS) was added to each well, and the plates were incubated for an additional 2 h at 37 °C in 5% CO_2_.

Fluorescence signals were then recorded using a Synergy HT microplate reader (BioTek Instruments, United States) with excitation and emission wavelengths set at 530/25 and 590/35 nm, respectively. The impact of the alkaloid fraction on cell viability was expressed as a percentage relative to untreated control cells. Two independent experiments were performed, each in triplicate.

### 2.10 Parameters of hemostasis

#### 2.10.1 Measurement of prothrombin time

Human plasma was incubated at 37 °C on a block heater. After incubation, the cuvette was transferred to the measuring holes. Hence, 100 μL of Dia-PT liquid (commercial preparation) was added. The PT was determined coagulometrically using an Optic Coagulation Analyzer, model K-3002 (Kselmed, Grudziadz, Poland), as per Malinowska et al. (2012).

#### 2.10.2 Measurement of thrombin time

Human plasma was added to a coagulometric cuvette and incubated at 37 °C on a block heater. Then, the cuvette was transferred to measuring holes, and 100 μL of thrombin (final concentration −5 U/mL) was added. The TT was determined coagulometrically using an Optic Coagulation Analyzer, model K-3002 (Kselmed, Grudziadz, Poland), as per Malinowska et al. (2012).

#### 2.10.3 Measurement of activated partial thromboplastin time

Human plasma was added to a coagulometric cuvette. Incubation was then conducted at 37 °C on a block heater with 50 μL of Dia-PTT liquid (commercial preparation). The cuvette was transferred to the measuring holes. Then, 50 μL of 25 mM CaCl_2_ was added. The APTT was determined coagulometrically using an Optic Coagulation Analyzer, model K-3002 (Kselmed, Grudziadz, Poland), as per Malinowska et al. (2012).

### 2.11 Data analysis

Statistical analyses were conducted using Statistica software version 10 (StatSoft). Data normality was evaluated using normal probability plots, while variance homogeneity was assessed by the Brown–Forsythe test. Differences among and between experimental groups were analyzed using one-way analysis of variance (ANOVA), followed by Duncan’s multiple comparison test. Statistical analysis was conducted using the Mann–Whitney test for samples with distributions deviating from normality in the comet assay. For clarity, only statistically significant differences between the tested preparations and the control or positive-control groups are reported.

Results are presented as the mean ± standard deviation (SD) or, for the comet assay, mean ± standard error of the mean (SEM). A *p-*value of less than 0.05 was considered statistically significant. To exclude outliers and uncertain data points, the Q-Dixon test was applied.

## 3 Results and discussion

In the current study, the structure of 11 new indole alkaloids was tentatively elucidated (Table 1), in addition to the known compounds from the various extracts of *R. stricta* leaves and stems that were detected (Tables 2 and 3). The analyses were achieved using the technique of electrospray ionization–quadrupole time-of-flight (ESI-Q-TOF) mass spectrometry in positive ionization mode [M + H]^+^ to explore fragmentation routes.

**TABLE 1:** Characterization of indole alkaloids first detected from *R. stricta* stem and leaf extracts using UPLC–ESI–MS/MS in positive ion mode [M + H]^+^.

Compound	R.t	MW	m/z [M + H]^+^	Major fragment (MS^n^)	Proposal molecular formula	Extract^*^			
Indole alkaloids from crude extract of stems (RS)						A	B	C	D
1	2.4	752	753	591 [M-162 + H]+, 429 [M-2X162 + H]^+^, 341 [M-2X162-88 + H]^+^, 215 [M-2X162-88–126 + H]^+^, 197 [M-2X162-88–126-18 + H]^+^	C_35_H_48_N_2_O_16_			+	
2	2.9	470	471	309 [M-162 + H]^+^, 307 [M-164 + H]^+^, 263 [M-164–44 + H]^+^	C_24_H_27_N_2_O_8_^−^		+		
3	4.2	780	781	601 [M-180(162 + 18)+H]^+^, 553 [M-228 + H]^+^, 391 [M-160–228-162 + H]^+^, 315 [M-180–48-162–76 + H]^+^, 229 [M-180–48-162–76-86 + H]^+^	C_37_H_52_N_2_O_16_			+	
4	5.7	366	367	351 [M-16 + H]^+^, 309 [M-16–42 + H]^+^	C_21_H_23_N_2_O_4_^−^	+			
5	6.25	368	369	355 [M-14 + H]^+^, 341 [M-2X14 + H]^+^, 297 [M-2X14-44 + H]^+^	C_22_H_28_N_2_O_3_	+			
6	6.33	368	369	355 [M-14 + H]^+^, 311 [M-14–44 + H]^+^, 297 [M-2X14-44 + H]^+^	C_22_H_28_N_2_O_3_	+			
7	10.7	696	697	349 [M-348 + H]^+^, 292[M-348–57+2H]^+^	C_42_H_40_N_4_O_6_				+

Indole alkaloids from the crude extract of leaves (RL)						A'	B'	C'	D'
8	2.4	752	753	591 [M-162 + H]^+^, 429 [M-2X162 + H]^+^, 341 [M-2X162-88 + H]^+^, 215 [M-2X162-88–126 + H]^+^, 197 [M-2X162-88–126-18 + H]^+^	C_35_H_48_N_2_O_16_			+	
9	2.6	546	547	367 [M-180 + H]^+^, 339 [M-180–28 + H]^+^, 296 [M-180–28-44 + H]^+^	C_28_H_38_N_2_O_9_		+		
10	7.8	430	431	297 [M-133+2H]^+^	C_24_H_34_N_2_O_5_		+		

+* = presence of these compounds in the extracts.

**TABLE 2:** Characterization of indole alkaloids previously detected from *R. stricta* stem crude extracts using UPLC–ESI–MS/MS in positive ion mode [M + H]^+^.

Rt (min)	Molecular formula	Compound name	MW	*m/z* [M + H]^+^	Major fragment	Extract*			
						A	B	C	D
4.9	C_21_H_24_N_2_O_4_	Rhazicine, rhazizine, or isorhazicine	368	369	299 [M-70 + H]^+^	+			
5.4	C_21_H_24_N_2_O_3_	Akuammidine, rhazinol (analog of strictamine), geissoschizine, polyneuridine, or tetrahydroalstonine	352	353	335 [M-18 + H]^+^, 317 [M-2X18 + H]^+^	+			
6.0	C_21_H_24_N_2_O_3_	Akuammidine, rhazinol (analog of strictamine), or tetrahydroalstonine	352	353	313 [M-40 + H]^+^, 299 [M-40–14 + H]^+^	+			
6.3	C_21_H_24_N_2_O_4_	Rhazicine, rhazizine, or isorhazicine	368	369	354 [M-14 + H]^+^, 341 [M-2X14 + H]^+^, 297 [M-2X14-44 + H]^+^	+			
6.6	C_21_H_22_N_2_O_3_	Leepacine isomer II	350	351	311 [M-40 + H]^+^, 281 [M-40–30 + H]^+^	+			
6.9	C_21_H_24_N_2_O_3_	Akuammidine, rhazinol (analog of strictamine), and tetrahydroalstonine	352						
	353	337 [M-16 + H]^+^, 297 [M-16–40 + H]^+^	+						
7.5	C_21_H_22_N_2_O_3_	Leepacine isomer III	350	351	269 [M-82 + H]^+^	+			
7.8	C_27_H_34_N_2_O_9_	Strictosidine	530	531	309 [M-222 + H]^+^, 283 [M-222–26 + H]^+^, 265 [M-222–26-18 + H]^+^	+			
9.9	C_42_H_56_N_4_O_4_	Tetrahydropresecamine	680	681	341 [M-340 + H]^+^, 313 [M-340–28 + H]^+^, 116[M-340–28-197 + H]^+^			+	
11.4	C_40_H_54_N_4_O_2_	16S, 16′-decarboxytetra-hydrosecamine	622	623	379 [M-244 + H]^+^, 312 [M-244–67 + H]^+^, 302 [M-244–67-10 + H]^+^		+		
11.9	C_40_H_54_N_4_O_2_	16S, 16‟-decarboxytetra-hydrosecamine	622	623	399 [M-224 + H]^+^, 349 [M-274 + H]^+^, 312 [M-274–37 + H]^+^, 283 [M-274–37-29 + H]^+^, 209 [M-274–37-29–74 + H]^+^, 175 [M-274–37-29–74-34 + H]^+^, 147 [M-274–37-29–74-34–28 + H]^+^		+		
12.4	C_21_H_20_N_2_O_3_	Strictisidine	348	349	317 [M-32 + H]^+^, 287 [M-32–30 + H]^+^, 126 [M-32–30-61 + H]^+^	+			
12.8	C_26_H_30_N_2_O_8_	Strictosidine lactam	498	499	470 [M-29 + H]^+^, 341 [M-29–129 + H]^+^, 338 [M-29–132 + H]^+^, 311[M-29–132-27 + H]^+^, 290 [M-29–132-27–21 + H]^+^, 147 [M-29–132-27–21-143 + H]^+^				+
13.1	C_40_H_54_N_4_O_2_	16S, 16′-decarboxy tetra-hydrosecamine	622	623	415 [M-208 + H]^+^, 312 [M-208–103 + H]^+^		+		
13.4	C_20_H_22_N_2_O_3_								
	Strictamine-N-oxide, stricticine, sewarine, (±)-vincadiformine	338	339	282 [M-57 + H]^+^	+				
13.6	C_21_H_28_N_2_O_2_								
	N-Methyleuconulam, dihydrosecodine, vicadine	340	341	312 [M-29 + H]^+^	+				
13.9	C_40_H_54_N_4_O_2_	Decarboxytetra-hydrosecamine	622	623	340 [M-283 + H]^+^, 332 [M-283–8+H]^+^, 312 [M-283-8–20 + H]^+^	+			
14.4	C_21_H_20_N_2_O_3_	Strictisidine	348	349	334 [M-15 + H]^+^, 283 [M-15–51 + H]^+^, 175 [M-15–51-108 + H]^+^, 116 [M-15–51-108–59 + H]^+^, 78 [M-15–51-108–59-38 + H]^+^	+			

+*, presence of these compounds in the extracts.

**TABLE 3:** Characterization of the known indole alkaloids previously detected from *R. stricta* leaf crude extracts using UPLC–ESI–MS/MS in positive ion mode [M + H]^+^.

Rt (min)	Molecular formula	Compound name	MW	*m/z* [M + H]^+^	Major fragment	Extracts*			
						A′	B′	C′	D′
2.4	C_24_H_32_N_2_O_5_	Aspidospermiose	428	429	394 [M-35 + H]^+^, 376 [M-35–16 + H]^+^, 359 [M-35–16-18 + H]^+^, 215 [M-35–16-18–144 + H]^+^, 197 [M-35–16-18–144-28 + H]^+^, 179 [M-35–16–2X18–144–28 + H]^+^, 151 [M-35–16–3X18–144–28 + H]^+^	+			
5.3	C_21_H_24_N_2_O_3_	Rhazine, strictamine analog, tetrahydroalstonine	352	353	335 [M-18 + H]^+^, 327 [M-26 + H]^+^		+		
6.0	C_19_H_26_N_2_O	Rhazidine, rhazidigenine, dihydrocorynantheol	298	299	259 [M-40 + H]^+^, 241 [M-40–18 + H]^+^, 201 [M-2X40-18 + H]^+^, 185 [M-2X40-18–16 + H]^+^, 175 [M-2X40-18–26 + H]^+^, 145 [M-2X40-18–56 + H]^+^, 127 [M-2X40–2X18-56 + H]^+^, 108 [M-2X40–2X18-16–59 + H]^+^	+			
6.0	C_21_H_24_N_2_O_4_	Rhazicine, rhazizine, isorhazicine	368	369	353 [M-16 + H]^+^, 313 [M-16–40 + H]^+^, 299 [M-16–40-14 + H]^+^, 273 [M-16–40-14–26 + H]^+^		+		
6.4	C_22_H_26_N_2_O_4_	2-methoxy-1,2-di hydorhazimine, 17- methoxy-1-17-dihydorhazimine	382	383	355 [M-28 + H]^+^, 281 [M-28–74 + H]^+^	+			
7.4	C_21_H_22_N_2_O_3_	Leepacine isomer I	350	351	323 [M-28 + H]^+^, 274 [M-28–76 + H]^+^	+			
7.7	C_27_H_34_N_2_O_9_	Strictosidine	530	531	471 [M-60 + H]^+^, 415 [M-60–56 + H]^+^, 383 [M-60–56-32 + H]^+^, 355 [M-60–56-32–28 + H]^+^, 323 [M-60–56–2X32–28 + H]+, 279 [M-60–56–2X 32–28-44 + H]^+^		+		
9.7	C_21_H_20_N_2_O_3_	Strictisidine	348	349	299 [M-50 + H]^+^		+		
10.0	C_40_H_46_NO_2_	Rhazisidine	614	615	413 [M-202 + H]^+^, 329 [M-202–84 + H]^+^, 308 [M-202–84-21 + H]^+^, 283 [M-202–84-21–25 + H]^+^,175 [M-202–84-21–25-108 + H]^+^,116 [M-202–84-21–25-108–59 + H]^+^			+	
10.1	C_40_H_48_N_4_O_2_	3, 14-dehydrorhazigine	616	617	473 [M-144 + H]^+^. 433 [M-144–40 + H]^+^, 389 [M-144–40-44 + H]^+^, 353 [M-144–40-44–36 + H]^+^, 327 [M-144–40-44–36-26 + H]^+^, 309 [M-144–40-44–36-26–19 + H]^+^, 207 [M-144–40-44–36-26–19-102 + H]^+^			+	
10.7	C_21_H_20_N_2_O_3_	Strictisidine	348	349	317 [M-32 + H]^+^, 287 [M-32–30 + H]^+^		+		
11.1	C_26_H_30_N_2_O_8_	Strictosidine lactam	498	499	345 [M-145 + H]+, 311 [M-145–43 + H]^+^, 309 [M-14–5+H]+, 283 [M-145–45-26 + H]+, 172 [M-145–45-26–111 + H]+, 116 [M-145–45-26–111-56 + H]^+^		+		
11.9	C_42_H_52_N_4_O_4_	Secamine, presecamine	676	677	428 [M-249 + H]^+^, 400 [M-249–28 + H]^+^, 339 [M-249–28-61 + H]^+^, 312 [M-249–28-61–27 + H]^+^			+	
12.0	C_42_H_54_N_4_O_4_	Dihydropresecamine, dihydrosecamine	678	679	428 [M-251 + H]^+^, 340 [M-251–88 + H]^+^, 124 [M-251–88-216 + H]^+^				+
12.1	C_42_H_54_N_4_O_4_	Dihydropresecamine, dihydrosecamine	678	679	429 [M-250 + H]+, 340 [M-250–89 + H]+, 337 [M-250–89-3+H]^+^			+	+
12.7	C_42_H_54_N_4_O_4_	Dihydropresecamine, dihydrosecamine	678	679	348[M-136–131-51–13 + H]^+^, 340 [M-136–131-51–21 + H]^+^, 281[M-136–131-132 + H]^+^, 147 [M-136–131-132–134 + H]^+^, 116 [M-136–131-132–134-31 + H]^+^			+	
13.4	C_42_H_52_N_4_O_4_	Secamine, presecamine	676	677	339 [M-338 + H]^+^			+	
13.5	C_42_H_54_N_4_O_4_	Dihydropresecamine, dihydrosecamine	678	679	422 [M-257 + H]^+^, 340 [M-257–82 + H]^+^,334 [M-257–88 + H]^+^				+
13.8	C_42_H_54_N_4_O_4_	Dihydropresecamine, dihydrosecamine	678	679	420 [M-259 + H]^+^, 340 [M-259–80 + H]			+	
13.9	C_42_H_54_N_4_O_4_	Dihydropresecamine, dihydrosecamine	678	679	422 [M-257 + H]^+^, 340 [M-257–82 + H]^+^,334 [M-257–88 + H]^+^			+	
14.0	C_42_H_56_N_4_O_4_	Tetrahydropresecamine	680	681	348 [M-333 + H]^+^, 341 [M-340 + H]^+^			+	

+*, presence of these compounds in the extracts.

### 3.1 Chemical characteristics of the plant preparations

Plant-based secondary metabolites are still reinvestigated for their toxic activity and for their quality and quantity in plants. Therefore, advanced chromatographic techniques are performed to qualify and quantify them in plants, such as LC-MS/MS (Stöckigt et al., 2002) and UPLC-MS/MS (Mahida et al., 2026; Fu et al., 2025; Youssif et al., 2024; Och et al., 2017; Nagy et al., 2023).

**FIGURE 1:**
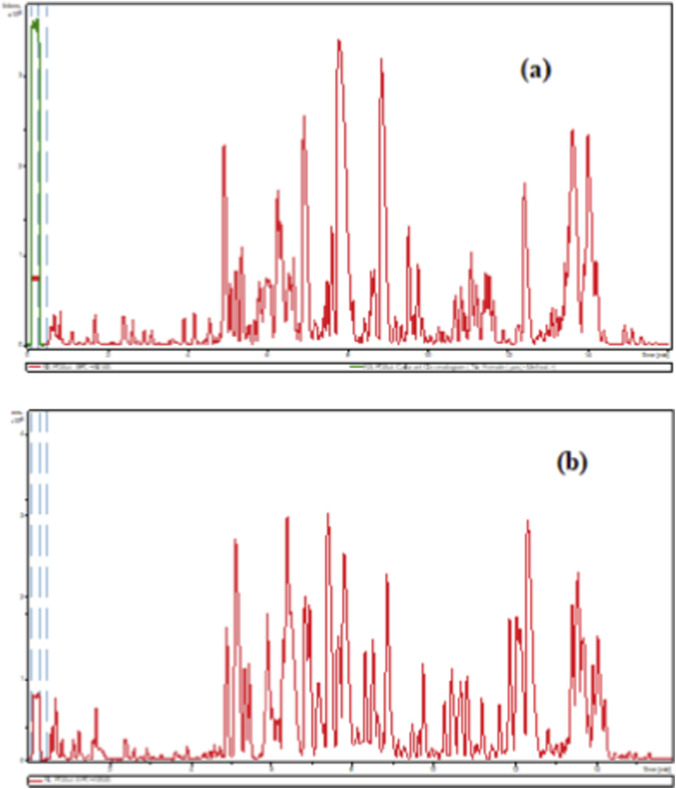
UPLC-ESI-MS/MS profile (positive ion mode) of the *R. stricta*: **(a)** Leaf extract (RL); **(b)** Stem extract (RS).

In our experiments, the established analytical method was performed to determine alkaloids in the stems (**RS**) and leaves (**RL**) of *R. stricta* and their extracts (Tables 1–3). The analysis of these samples revealed the existence of several indole alkaloids. These alkaloids were identified based on the detection of their maxima and the shape of the spectra. Exemplary UPLC chromatograms of **RS** and **RL** extracts are shown in Figures 1a,b.

### 3.2 Identification of the new indole alkaloids from the stem parts of R. stricta

Positive-mode electrospray ionization [M + H]^+^ can display enhanced ion responses for indole alkaloids as a result of the presence of a free couple of electrons on the nitrogen atom, which leads to ease of protonation, and so, this method was adopted in this study (Hamed et al., 2022; Pan et al., 2015). The use of a UPLC-C18 column displayed a satisfactory resolution of indole alkaloids within 16 min. ESI–MS spectra were acquired in positive ion mode [M‏+H]^+^, which yielded the best results in the MS/MS experiment. [M‏+H]^+^ ions were recognized for all the analyzed signals and underwent diverse fragmentation approaches; a comparison between the MS/MS spectra led to complementary structural knowledge and allowed a good realization of the fragmentation patterns of these metabolites. Daughter ions were recognized for all the analyzed peaks and were subject to various fragmentation methods. A comparison between the MS/MS spectra led to accurate structural knowledge and allowed a great realization of the fragmentation profiles of these secondary metabolites.

**FIGURE 2:**
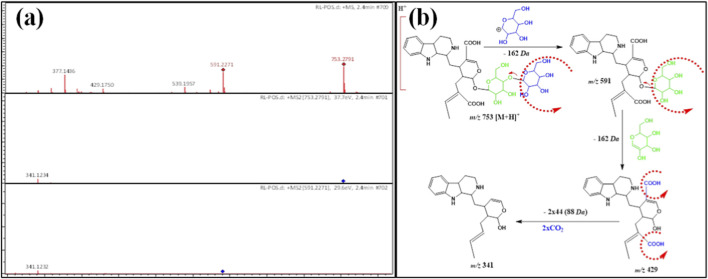
**(a)** ESI–MS spectra of compounds 1 and 8 at *m/z* 753 [M + H]^+^. **(b)** Proposal fragmentation pattern and structure of compounds 1 and 8.

ESI-MS/MS of compounds **1** and **8** showed the protonated ion signal at *m/z* 753 [M + H]^+^ at Rt 2.4 min, identical to the molecular formula of C_35_H_48_N_2_O_16_. The protonated product ion at *m/z* 591 [M-162 + H]^+^ was due to the elimination of the outer hexose moiety. In addition, the detected protonated ion product at *m/z* 429 M-2x162 + H]^+^ was attributed to cleavage of another hexose moiety. The diagnostic product ion with 429 *amu* was more correlated with the indole alkaloidal glycoside strictosidine (Akhgari et al., 2015a; Akhgari et al., 2015b) but differed from it by possessing two carboxylic groups, which was clear by the cleavage of 88 *amu* (2 × 44 *Da*) (Table 1; Figures 2a,b). From the above data, compounds **1** and **8** from *R. stricta* stem **(RS)** and leaf **(RL)** have not been detected from *R. stricta* and were tentatively named **rhazyaosidine A**.

**FIGURE 3:**
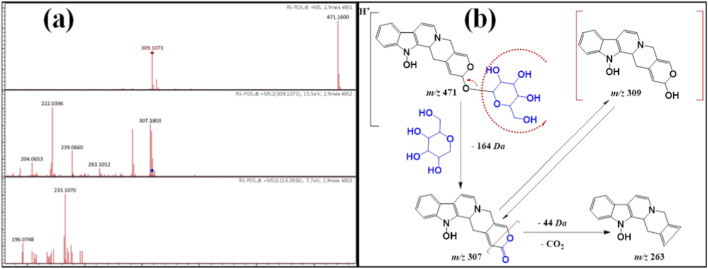
**(a)** ESI–MS spectra of compound 2 at *m/z* 471 [M + H]^+^. **(b)** Proposal fragmentation pattern and structure of compound 2.

Compound **2** showed a protonated ion peak at *m/z* 471[M + H]^+^ at Rt. 2.9 min, identical to the molecular formula C_24_H_27_N_2_O_8_^−^ (Table 1). This molecular formula indicated that compound **3** was an N-oxide indole alkaloid. Its MS/MS spectrum exhibited a diagnostic protonated product ion at *m/z* 309 [M-162 + H]^+^, which was due to the splitting of the hexose moiety. This ion product was a ketonic form of the aglycone, which was more stable. Furthermore, the enolic form gave a protonated ion fragment at *m/z* 307 [M-164 + H]^+^, which was less stable than the ketonic form. Hence, the ketonic form fragmented into the product ion at *m/z* 263 [M-162–44 + H]^+^, attributed to cleavage of the carbon dioxide molecule (Figures 3a,b). From all the above data, compound **3** was first recorded from *R. stricta* and named **rhazyaosidine B**.

**FIGURE 4:**
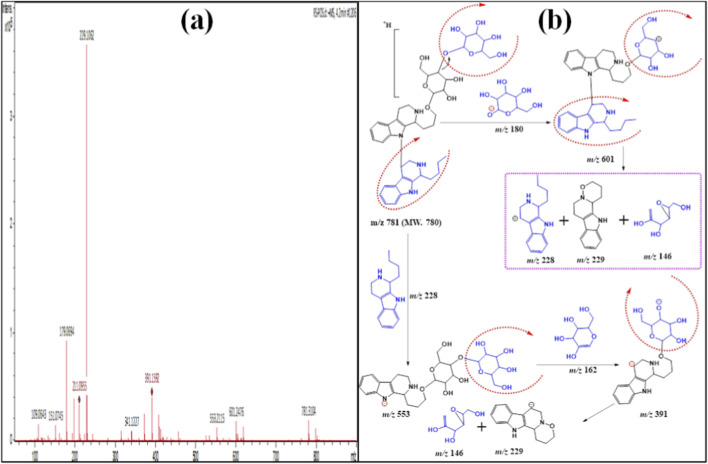
**(a)** ESI–MS spectra of compound 3 at *m/z* 781 [M + H]^+^. **(b)** Proposal fragmentation pattern and structure of compound 3.

Table 1 displays the protonated ion signal at *m/z* 781 [M + H]^+^ at Rt. 4.2 min for compound **3,** identical to the formula C_37_H_52_N_2_O_16_. Its ESI-MS/MS shows a diagnostic daughter ion at *m/z* 601 [M-180 + H]^+^, due to cleavage of one moiety of hexose and 18 *Da* (H_2_O). Figures 4a and b exhibit the cleavage of C_15_H_19_N_2,_ and hence an ion product was detected at *m/z* 553 [M-228 + H]^+^. Moreover, the protonated product ion at *m/z* 391 [M-228–162 + H]^+^ was due to the splitting of one hexose moiety and an indole alkaloid derivative. ESI-MS/MS displayed a diagnostic ion product at *m/z* 229 [M-228–2x162 + H]^+^ due to cleavage of another hexose moiety. This indicated the presence of another indole alkaloid derivative. The product ion peaks at *m/z* 553 and at *m/z* 229 indicated the presence of two conjugated alkaloidal moieties. Moreover, the ion fragment at *m/z* 229 [M-391–2x162–228 + H]^+^ was linked with two hexose moieties. The presence of the ion fragment with 229 *amu* hydroxyl group was substituted on the D ring (Figures 4a and b). From the above observed data, compound **3** was similar to hydroxylyohimbine alkaloid derivatives, which was proven by the existence of the ion product with a 229 *amu* hydroxyl group substituted on the D or E ring (Kumar et al., 2016). Compound **3** was first recorded from *R. stricta* and named **rhazyaosidine C**.

**FIGURE 5:**
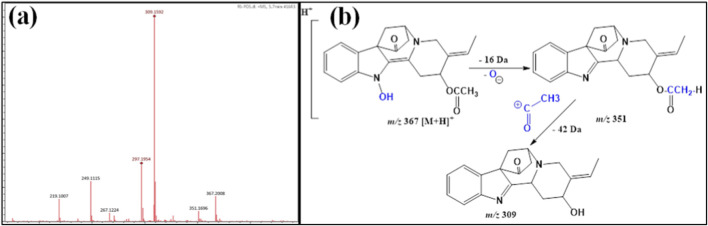
**(a)** ESI–MS spectra of compound 4 at *m/z* 367 [M + H]^+^. **(b)** Proposal fragmentation pattern and structure of compound 4.

Compound **4** showed an ion peak at *m/z* 367 [M + H]^+^ at a retention time of 5.7 min, identical to the molecular formula (Table 1). It exhibited a diagnostic protonated daughter ion at *m/z* 351 [M-16 + H]^+^ due to the cleavage of an oxygen atom, indicating that compound **4** was an N-oxide indole alkaloid derivative. Compound **4** resembled leepacine isomers that were isolated from *R. stricta* (Chaudhary and Al-Jowaid, 1999; Bukhari et al., 2017), but there were two differences: compound **4** contains an N-oxide feature, and it contains an acetoxy group instead of methyl acetate, which leepacine isomers have (Figures 5a and b). This was cleared by the existence of a protonated ion signal at *m/z* 309 [M-16–42 + H]^+^. The presence of a daughter ion at *m/z* 309 [M-58 + H]^+^ was due to splitting of one acetoxy moiety (42 *amu*) (Figures 5a,b). From the above date, compound **4** was first recorded from *R. stricta* and was given the name **arafacine**.

**FIGURE 6:**
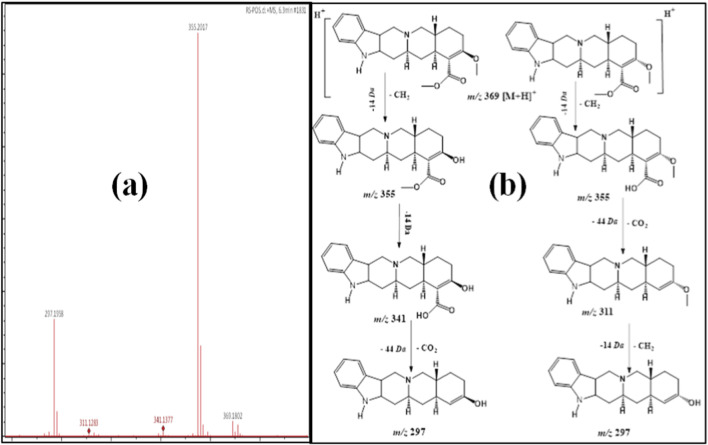
**(a)** ESI–MS spectra of compounds 5 and 6 at *m/z* 369 [M + H]^+^. **(b)** Proposal fragmentation pattern and structure of compounds 5 and 6.

Compounds **5** (Rt 6.3 min) and **6** (Rt 6.5 min) showed exact protonated ion signals at *m/z* 369 [M + H]^+^ identical to the molecular formula C_22_H_28_N_2_O_3_ (Table 1). Their first ESI-MS/MS displayed the same diagnostic protonated daughter ion at *m/z* 355 [M-14 + H]^+^, which was attributed to the splitting of 14 *amu* correlated to the splitting of one methylene unit (Table 1). The product ions with 355 *amu* were similar to yohimbine and yohimbine isomer (Akhgari et al., 2015a; Akhgari et al., 2015b). Their second and third steps of fragmentation were different: one lost a second 14 *amu* for the methylene moiety (*m/z* 341 [M-2x14 + H]^+^, compound **6**), and the other lost 44 *amu* as a result of the splitting of the CO_2_ moiety (*m/z* 311 [M-44–14 + H]^+^, compound **7**). The third steps for both were alternated, where compound 5 lost 44 *amu* and compound 6 lost 14 *amu* (Figures 6a,b). From the above date, compounds **5** and **6** were first recorded from *R. stricta* and were tentatively assigned as methyl-yohimbine and its isomer.

**FIGURE 7:**
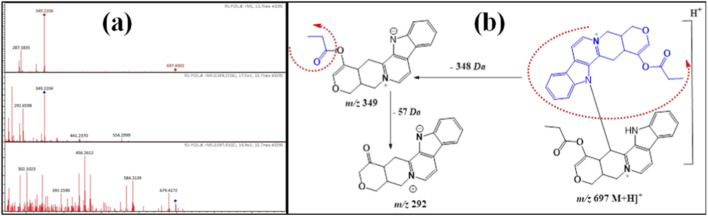
**(a)** ESI-MS spectra of compound 7 at *m/z* 697 [M + H]^+^. **(b)** Proposal fragmentation pattern and structure of compound 7.

Compound **7** exhibited a protonated ion peak at *m/z* 697 [M + H]^+^ at Rt 10.7 min, identical to the molecular formula C_42_H_40_N_4_O_6_^+^. The existence of a diagnostic daughter ion signal at *m/z* 349 [M-348 + H]^+^ indicated that compound **7** was a dimer of the monomer serpentine (Akhgari et al., 2015a; Akhgari et al., 2015b). In addition, its ESI-M/MS spectrum exhibited a protonated product ion at *m/z* 292 [M-348–57+2H]^+^ due to the cleavage of the CO_2_CH_2_ moiety (Figures 7a,b). From the above data, compound **7** was first recorded from *R. stricta* and was tentatively assigned as a serpentine dimer.

**FIGURE 8:**
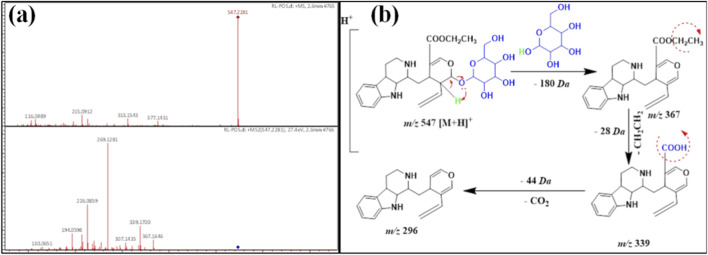
**(a)** ESI-MS spectra of compound 9 at *m/z* 547 [M + H]^+^. **(b)** Proposal fragmentation pattern and structure of compound 9.

Compound **9** showed a protonated ion signal at *m/z* 547 [M + H]^+^ with Rt at 2.6 min, identical to the formula C_28_H_38_N_2_O_9_ (Table 1). Its MS/MS fragmentation pattern displayed a diagnostic daughter ion signal at *m/z* 376 [M-180(162 + 18)+H]^+^, referred to as the splitting of one hexose moiety and 18 *amu*. Moreover, the daughter ion peak at *m/z* 339 [M-180(162 + 18)-28 + H]^+^ was due to the splitting of the ethylene moiety and the presence of 339 *amu,* indicating the presence of a strictosidine derivative (Akhgari et al., 2015a; Akhgari et al., 2015b). This was approved by the presence of the product ion at *m/z* 296 [M-180–28-44 + H]^+^, which was due to the splitting of the carbon dioxide molecule (44 *amu*). Compound **9** was similar to strictosidine except for the presence of –COOCH_2_CH_3_ instead of –COOCH_3_ (Figures 8a,b). Hence, compound **9** was detected for the first time in *R. stricta* and given the name **rhazyaosidine D**.

Based on the recorded maxima, the fragmentation profile of the spectra, the molecular weights, and the fragmentation patterns of the known metabolites, our data were successfully used to discriminate the remaining detected indole alkaloids

The known compounds were identified by comparing their spectroscopic results with those in the literature (Tables 2 and 3) (Ahmed et al., 2018; Pan et al., 2015; Kumar et al., 2016).

### 3.3 Effect of different extracts from the leaf and stem of *R. stricta* on oxidative stress *in vitro*

Oxidative stress is attributed to the excessive production of free radicals, which leads to lipid peroxidation, protein carbonylation, the oxidation of thiols, and DNA damage. On the other hand, various phytochemicals often have antioxidant potential. Although various *R. stricta* preparations have special health-promoting properties (Albeshri et al., 2021), their biological activities at the molecular level remain only partially understood, and scientific research on the bioactive compounds, including alkaloids, of this plant is still limited.

Alkaloids exhibit antioxidant activity within a defined therapeutic window, representing the exposure range in which they reduce oxidative stress without triggering cytotoxic or pro-oxidant effects. At lower, physiologically compatible levels, alkaloids contribute to free radical scavenging and redox balance, as demonstrated in studies showing their role in mitigating oxidative stress within herbal medicines and phytochemical mixtures. Reviews of alkaloid antioxidant activity confirm that compounds such as berberine, quinine derivatives, and caffeine display measurable antioxidant capacity in chemical assays (e.g., DPPH, FRAP), indicating that beneficial effects occur within a controlled lower exposure range (Halliwell, 2024). However, as exposure increases beyond the optimal range, many alkaloids, especially those with strong pharmacological actions, begin to exhibit cytotoxic, pro-oxidant, or pathway-disruptive effects. This is consistent with reports highlighting the potent bioactivity of alkaloids used in anticancer, antimicrobial, and neuroactive contexts, where higher doses can induce apoptosis or disturb mitochondrial function. Because of these strong biological effects, the upper boundary of the therapeutic window is quickly reached, and alkaloids may shift from antioxidant protection to cellular stress or toxicity (Gulcin, 2025). In summary, antioxidant alkaloids act beneficially only within a moderate and compound-specific therapeutic window. This dose-dependent duality underscores the need for careful characterization of safety margins when evaluating alkaloids as potential antioxidant agents.

**FIGURE 9:**
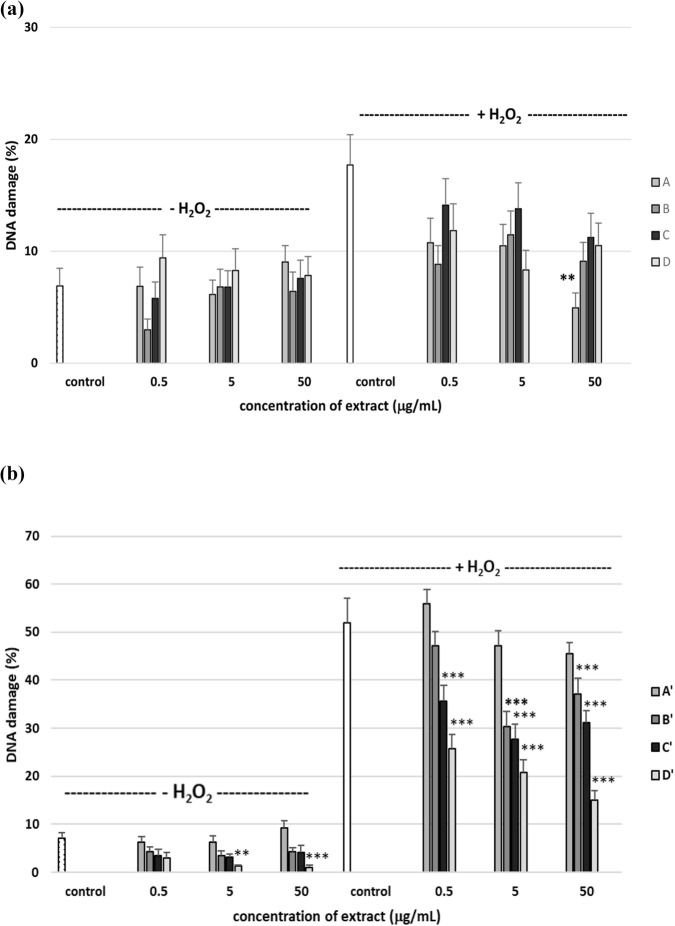
Effects of the four *R. stricta* stem (A–D, concentration range 0.5–50 μg/mL, pre-incubation time: 2 h **(a)**) and leaf (A′–D′, concentration range 0.5–50 μg/mL, pre-incubation time: 2 h **(b)**) extracts on DNA damage in PBMCs treated with 20 µM H_2_O_2_ (incubation time: 15 min on ice). Results presented as the mean ± SEM (n = 100). Mann–Whitney test: **p < 0.01, ***p < 0.001 compared with control (PBMCs not treated with plant extract).

**FIGURE 10:**
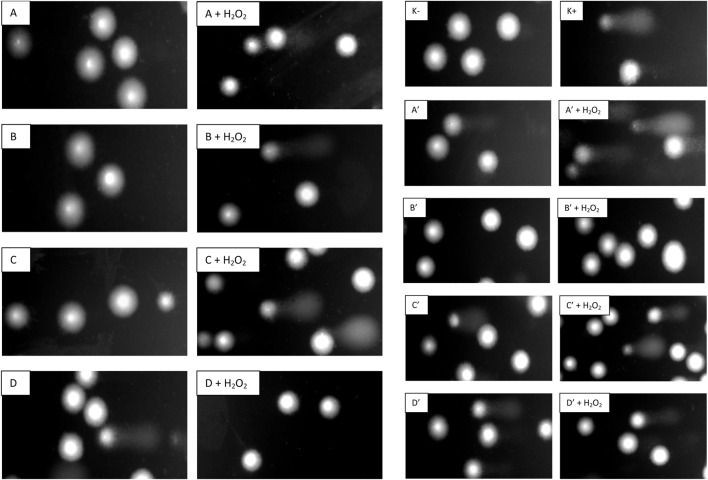
Representative photos of comets obtained in the alkaline version of the comet assay after pre-incubation of PBMCs with *R. stricta* stem **(A–D)** and leaf extracts **(A′–D′)** at 5 μg/mL and incubation with 20 µM H_2_O_2_ for 15 min on ice. K−, negative control (untreated PBMCs); K+, positive control (PBMCs incubated with H_2_O_2_ at 20 µM for 15 min on ice).

Our study is the first to comprehensively assess the effect of the four *R. stricta* stem (A–D) and leaf (A′–D′) extracts on the level of selected parameters of oxidative stress in plasma and PBMCs, employing *in vitro* experimental systems related to the blood physiology and cardiovascular system. Exposure of PMBCs to an oxidant, H_2_O_2_, resulted in a significantly enhanced level of DNA damage. We observed a significant difference in the level of DNA damage induced by H_2_O_2_ in the experiment with extracts A–D and A′–D′ (17.71% vs*.* 51.89%). This difference is because PBMCs from two different buffy coat cells were used for the experiments (Figures 9a,b). As shown in Figure 9a, not all A–D extracts induced DNA damage in PBMCs. These extracts also did not reduce DNA fragmentation induced by H_2_O_2_, except for extract A at 50 μg/mL (*p* < 0.01). In the case of extracts A′–D′, we observed a reduction of endogenous DNA damage after incubation with extract D′ at the concentrations of 5 μg/mL (*p* < 0.01) and 50 μg/mL (*p* < 0.001). In addition, we observed a strong inhibition of DNA oxidative damage by extracts B′, C′, and D′ at concentrations of 5 μg/mL and 50 μg/mL (*p* < 0.001). After incubation with extracts C′ and D′ at 0.5 μg/mL, we also observed a reduction in the level of DNA damage (*p* < 0.001). Figure 10 shows pictures of comets from cells pre-incubated with A–D and A′–D′ in which a reduction of DNA oxidative damage was observed (Figure 10).

In another study, human lymphocyte cultures were exposed to aqueous leaf extract of *R. stricta* at concentrations of 6, 12, and 24 g/L for 24, 48, and 72 h (Baeshen et al., 2009). Cytogenetic analysis revealed a significant, concentration- and time-dependent reduction in the mitotic index. Various cellular and nuclear abnormalities were observed, including increased proportions of interphase cells, elevated micronuclei formation, chromosome stickiness, colchicine-induced metaphases, and binucleated cells. Microscopic examination indicated extensive necrosis in treated cells at all tested concentrations, suggesting potential anticancer activity. Comet assay results consistently demonstrated DNA damage that increased with both concentration and exposure time. Collectively, these findings indicate that aqueous *R. stricta* leaf extract exhibits mutagenic, clastogenic, and potentially anticancer effects in human lymphocytes *in vitro* (Baeshen et al., 2009).

Furthermore, whole aqueous and alkaloid fractions but not non-alkaloid extracts of *R. stricta* were shown to alter genomic RAPD (random amplified polymorphic DNA) profiles, induce significant DNA damage, increase micronucleus frequency, promote chromosomal aberrations, and reduce the mitotic index across all tested doses. These data suggest that oral administration of *R. stricta* extracts induces genotoxic and clastogenic effects in rat (*Rattus norvegicus*) leukocytes (Baeshen et al., 2014).

**FIGURE 11:**
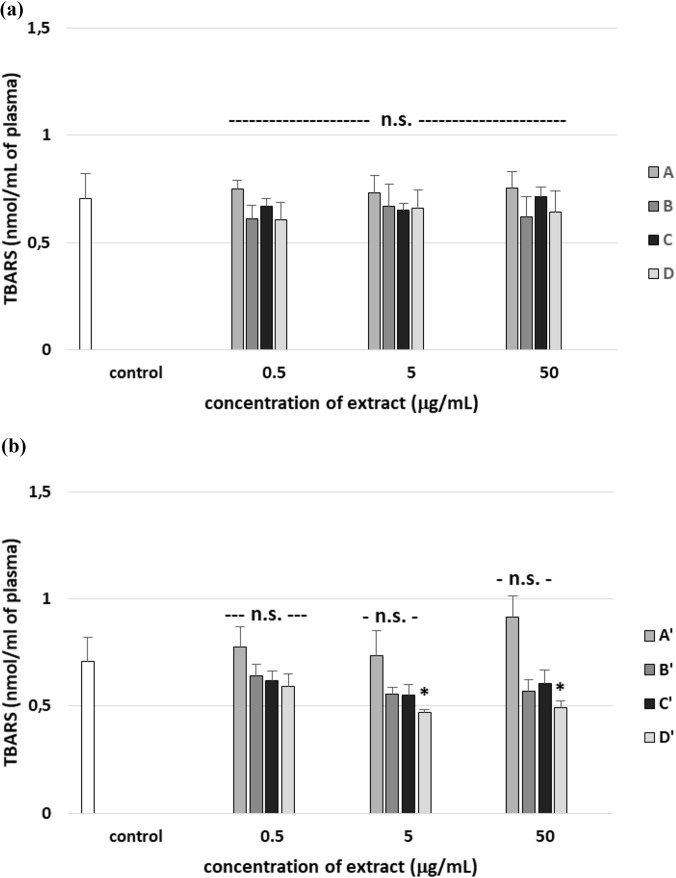
Effects of the four *R. stricta* stem extracts (A–D, concentration range 0.5–50 μg/mL, incubation time: 30 min; **(a)**) and leaf extracts (A′–D′, concentration range 0.5–50 μg/mL, incubation time: 30 min; **(b)**) on lipid autoperoxidation in plasma. Results were given as the mean ± SD (n = 6). One-way ANOVA, followed by multicomparison Duncan’s test: n.s, *p* > 0.05; *p < 0.05 compared with the control (plasma not treated with plant extract).

**FIGURE 12:**
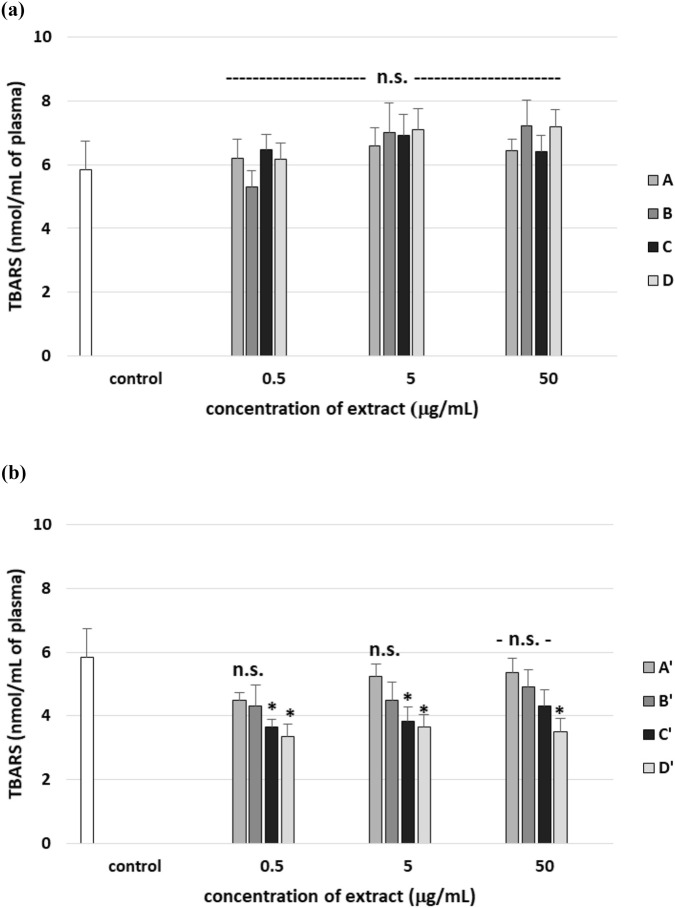
Effects of the four *R. stricta* stem extracts (A–D, concentration range 0.5–50 μg/mL, pre-incubation time: 5 min; **(a)**) and leaf extracts (A′–D′, concentration range 0.5–50 μg/mL, pre-incubation time: 5 min; **(b)**) on lipid peroxidation in plasma treated with H_2_O_2_/Fe^2+^ (incubation time – 25 min). Results were given as the mean ± SD (n = 6). One-way ANOVA, followed by multicomparison Duncan’s test: n.s, *p* > 0.05; *p < 0.05, compared with the control (plasma treated with H_2_O_2_/Fe^2+^ and without plant extract).

The oxidative stress model based on the H_2_O_2_/Fe^2+^ system is widely used, but plasma is a complex biological matrix that contains endogenous antioxidants and metal-binding proteins (e.g., albumin, transferrin). These components may influence the efficiency of the Fenton reaction and the formation of hydroxyl radicals. However, we have also noted that exposure of human plasma to H_2_O_2_/Fe^2+^ resulted in a significantly enhanced level of lipid peroxidation, protein carbonylation, and oxidation of protein thiols. On the other hand, no positive effects were observed in all tested A–D stem extracts on plasma lipid autoperoxidation (Figure 11a). However, leaf extract D’ (at two used concentrations: 5 and 50 μg/mL) was found to protect human plasma against lipid autoperoxidation (Figure 11b), but extracts A′, B′, and C′ did not exert activity. Moreover, all tested extracts from *R. stricta* stems did not change the level of lipid peroxidation in plasma treated with H_2_O_2_/Fe^2+^ (Figure 12a). As demonstrated in Figure 12B, two tested extracts from *R. stricta* leaf (C′ and D′, at all used concentrations) were found to protect plasma against H_2_O_2_/Fe^2+^—induced lipid peroxidation. Extract D′ exerted the strongest effect at the highest dose (50 μg/mL) (Figure 12b).

**FIGURE 13:**
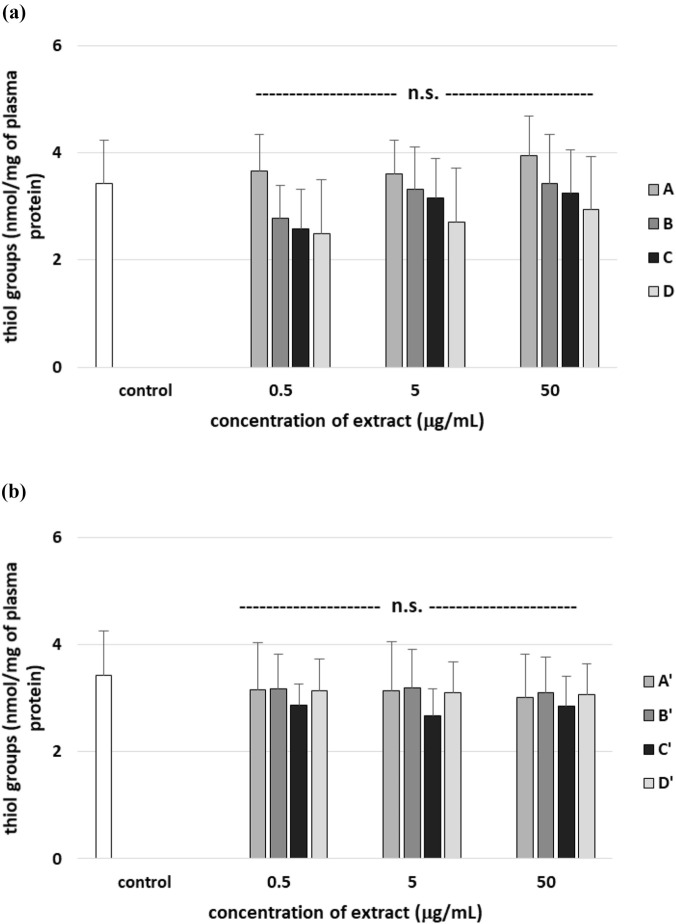
Effects of the four *R. stricta* stem extracts (A–D, concentration range 0.5–50 μg/mL, incubation time: 30 min; **(a)**) and leaf extracts (A′–D′, concentration range 0.5–50 μg/mL, incubation time: 30 min; **(b)**) on the level of thiol groups in plasma proteins. Results were given as the mean ± SD (n = 6). One-way ANOVA, followed by multicomparison Duncan’s test: n.s, *p* > 0.05; *p < 0.05, compared with the control (plasma not treated with plant extract).

**FIGURE 14:**
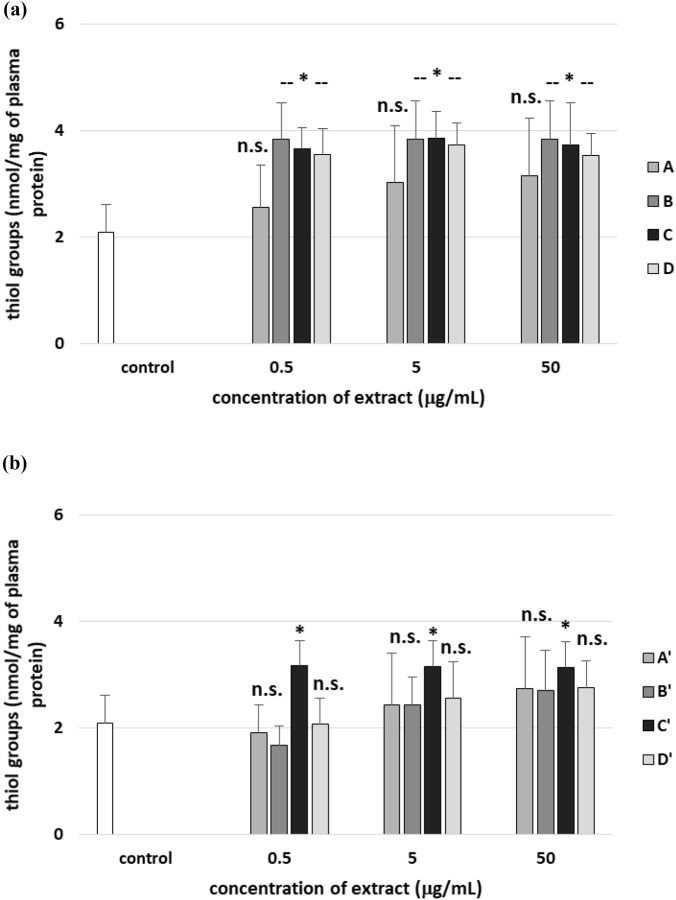
Effects of the four *R. stricta* stem extracts (A–D, concentration range 0.5–50 μg/mL, incubation time: 30 min; **(a)**) and leaf extracts (A′–D′, concentration range 0.5–50 μg/mL, incubation time: 30 min; **(b)**) on the oxidative damages of plasma protein treated with H_2_O_2_/Fe^2+^—the level of thiol groups in plasma proteins. Results were given as the mean ± SD (n = 6). One-way ANOVA, followed by multicomparison Duncan’s test: n.s, *p* > 0.05; *p < 0.05, compared with the control (plasma treated with H_2_O_2_/Fe^2+^ and without plant extract).

After 30 min incubation of human plasma with all tested extracts from *R. stricta* stem and leaf, the number of thiol groups in plasma proteins did not significantly change compared to control (plasma without H_2_O_2_/Fe^2+^) (Figures 13a,b). On the other hand, three tested extracts from *R. stricta* stem (B, C, and D, at all used concentrations of 0.5–50 μg/mL) significantly increased the level of thiol groups in plasma treated with H_2_O_2_/Fe^2+^ compared to control (plasma treated with H_2_O_2_/Fe^2+^) (Figure 14a). Moreover, the effect of extract A from *R. stricta* stem and extracts A′, B′ and C’ from *R. stricta* leaf at all used concentrations was not statistically significant (Figures 14a,b). Only the C′ extract from *R. stricta* leaf significantly increased the level of thiol groups in human plasma treated with H_2_O_2_/Fe^2+^ compared to control (with H_2_O_2_/Fe^2+^) (Figure 14b).

**FIGURE 15:**
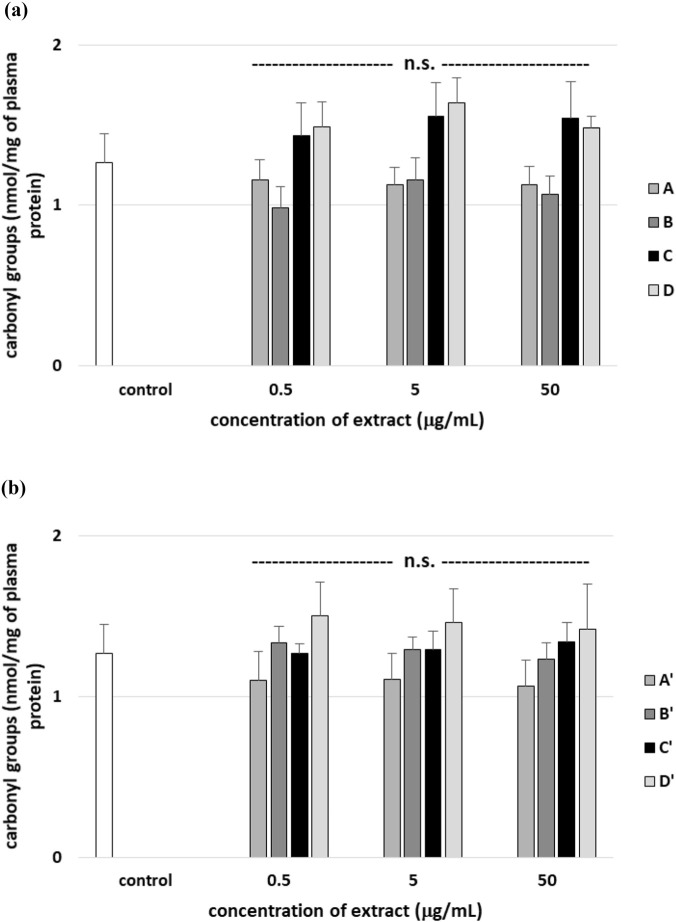
Effects of the four *R. stricta* stem extracts (A–D, concentration range 0.5–50 μg/mL, incubation time: 30 min; **(a)**) and leaf extracts (A′–D′, concentration range 0.5–50 μg/mL, incubation time: 30 min; **(b)**) on the level of carbonyl groups in plasma proteins. Results were given as the mean ± SD (n = 6). One-way ANOVA, followed by multicomparison Duncan’s test: n.s, *p* > 0.05; *p < 0.05, compared with the control (plasma not treated with plant extract).

**FIGURE 16:**
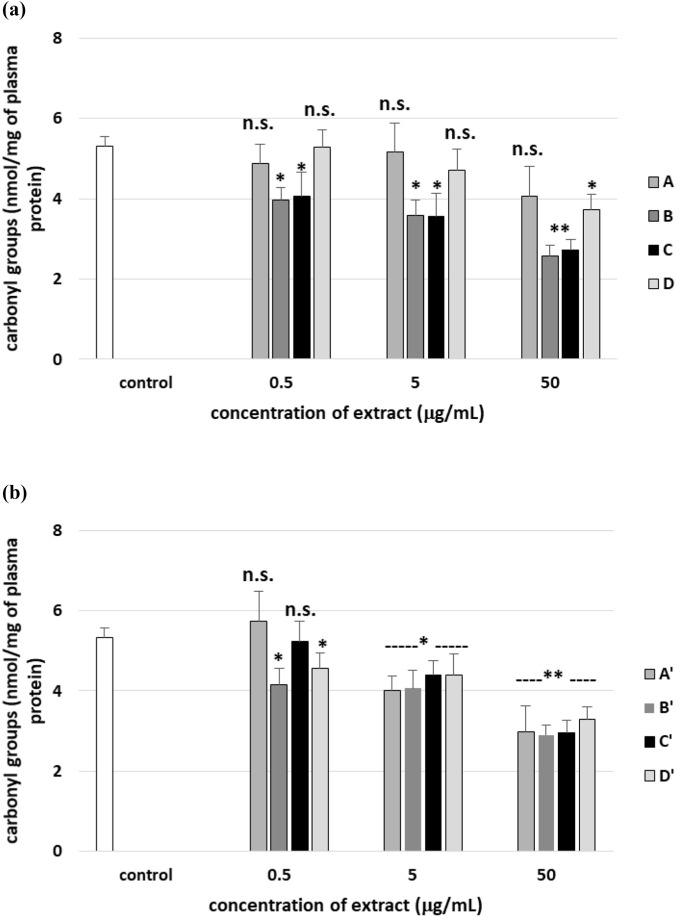
Effects of the four *R. stricta* stem extracts (A–D, concentration range 0.5–50 μg/mL, incubation time: 30 min; **(a)**) and leaf extracts (A′–D′, concentration range 0.5–50 μg/mL, incubation time: 30 min; **(b)**) on the oxidative damages of plasma protein treated with H_2_O_2_/Fe^2+^—the level of carbonyl groups in plasma proteins. Results were given as the mean ± SD (n = 6). One-way ANOVA, followed by multicomparison Duncan’s test: n.s, *p* > 0.05; *p < 0.05 and **p < 0.01 compared with the control (plasma treated with H_2_O_2_/Fe^2+^ and without plant extract).

Figures 15a and b demonstrate that all tested leaf (A′–D′) and stem (A–D) extracts do not statistically significantly change the number of carbonyl groups in plasma proteins. However, two used extracts from *R. stricta* stem (B and C, at all used concentrations: 0.5–50 μg/mL) significantly decreased the level of carbonyl groups in plasma proteins treated with H_2_O_2_/Fe^2^, compared to control (plasma treated only with H_2_O_2_/Fe^2+^) (Figure 16a). These effects on this process were dose-dependent (Figure 16a). Four used leaf extracts (at two high tested concentrations: 5 and 50 μg/mL) also reduced the level of carbonyl groups in plasma treated with H_2_O_2_/Fe^2+^ compared to control plasma (only treated with H_2_O_2_/Fe^2+^) (Figure 16b).

Mechanistically, many natural indole alkaloids influence mitochondrial integrity by modulating regulated cell death (RCD) pathways, including apoptosis, autophagy, necroptosis, and ferroptosis, which are closely linked to mitochondrial membrane stability and ROS flux. Studies summarize how clinically relevant indole alkaloids such as vincristine, vinblastine, and staurosporine regulate mitochondrial signaling nodes, thereby influencing membrane potential, cytochrome c release, and oxidative cascades central to cell survival or death. By modulating these pathways, indole alkaloids can either protect mitochondrial membranes under controlled redox conditions or induce membrane destabilization when shifting toward cytotoxic or pro-oxidant activity (Qin et al., 2022).

The antioxidant mechanisms of all tested extracts from *R. stricta* leaf and stem (observed in various biological models, including human plasma) may include scavenging oxidants (H_2_O_2_ itself and H_2_O_2_/Fe^2+^—derived radicals—^•^OH (one of the most aggressive reactive oxygen species)). In addition, a novel important aspect of our findings is that all tested stem and leaf extracts differed in terms of antioxidant activity in an experimental *in vitro* model based on crucial elements of hemostasis, including human plasma. The easiest way to explain these differences relates to the different chemical profiles of each of the extracts used. Other research has also noted that extracts from various parts of *R. stricta* possess antioxidant activity, which is in line with our present results. However, there are only a few studies on the antioxidant potential of *R. stricta* (Ali et al., 2000; Iqbal et al., 2019) which sometimes do not have the phytochemical characteristics of *R. stricta* preparations. For example, Iqbal et al. (2019) studied the antioxidant potential of different extracts (water, 80% methanol, 70% ethanol, and diethyl ether) from *R. stricta* leaf in the linoleic acid system, metal chelating activity, reducing power, and other parameters (in an *in vitro* model). They observed that the methanolic extract has the highest total phenolic content and antioxidant properties among the tested extracts. Ali et al. (2000) also noted the antioxidant action of extract from *R. stricta* leaves (0.25, 1.0, and 4.0 g/kg/day, for 3 days) in rats. At a dose of 1.0 g/kg, *R. stricta* extract significantly increased the level of glutathione in the liver. In addition, at the higher dose (4.0 g/kg), the tested plant extract decreased lipid peroxidation (measured by TBARS). The antioxidant properties of *R. stricta* root fractions were also identified, with an IC_50_ of 400–776 g/mL (Mahmood et al., 2020).

### 3.4 Effect of different extracts from *R. stricta* leaf and stem on the cytotoxicity of PBMCs

**FIGURE 17:**
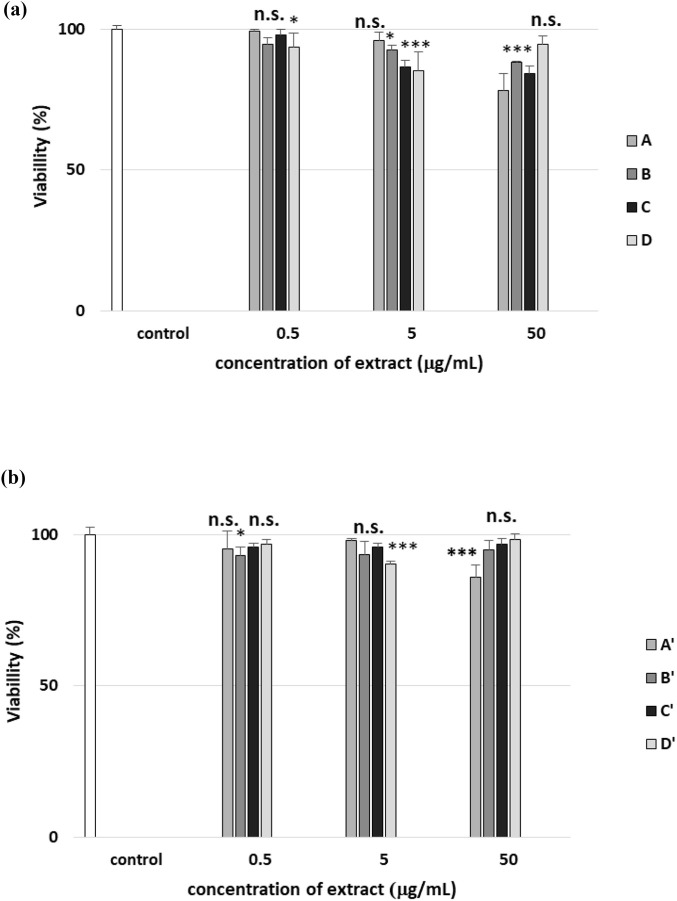
Effect of the four *R. stricta* stem extracts (A–D, concentration range 0.5–50 μg/mL) **(a)** and leaf extracts (A′–D′, concentration range 0.5–50 μg/mL) **(b)** on the viability of PBMCs. Cell viability was measured after 24 h incubation with plant extracts. Data were represented as the means ± SD of (n = 6). Test: ANOVA: n.s, *p* > 0.05; *p < 0.05 and ***p < 0.001 compared with the control (PBMCs not treated with plant extract).

Indole alkaloids belong to the group of biologically active compounds, and their broad spectrum of potential impact on cell metabolism generates much interest among researchers. However, the main limiting factor of the biological activity of the secondary metabolite compounds is their poor bioavailability. Moreover, the metabolism of indole alkaloids may change their cytotoxic activity. Therefore, not only the bioavailability but also the toxicity of phytochemicals, including alkaloids, is an important element in the evaluation of their biological potential. In our present study, the cytotoxicity of *R. stricta* stem (A–D) and leaf (A′–D′) extracts, each containing indole alkaloids in PBMCs, was examined in the concentration range of 0.5–50 μg/mL. We measured the viability of PBMCs after 24 h of incubation with extracts A–D and A′–D′ (Figures 17a,b). The results indicate that extracts isolated from *R. stricta* stems(A–D) are more cytotoxic than *R. stricta* leaf extracts (A′–D′). After incubating PBMCs with 50 μg/mL stem extracts, we observed a decrease in cell viability for extracts A, B, and C (*p* < 0.001). In the case of leaf extracts at 50 μg/mL, we noticed a decrease in cell viability only for extract A’ (*p* < 0.001) (Figure 17b).

Other studies have shown that extracts derived from different tissues of *R. stricta* display cytotoxic properties, with a stronger effect on cancer cells than on normal cells (Elkady, 2013). It has been reported that a crude alkaloid extract from *R. stricta* (CAERS) markedly suppressed the proliferation of non-small cell lung cancer (NSCLC) A549 cells in both a time- and dose-dependent manner. Moreover, cytotoxicity analyses revealed a synergistic interaction between CAERS and the chemotherapeutic agent cisplatin, resulting in the enhanced inhibition of A549 cell growth. Notably, CAERS did not exert a significant cytotoxic effect on non-cancer human fibroblasts (HF-5).

The growth-inhibitory effect of CAERS on A549 cells was associated with the induction of apoptosis, as evidenced by characteristic morphological alterations, DNA fragmentation, an elevated Bax/Bcl-2 ratio, mitochondrial cytochrome c release, the activation of caspases-3 and -9, and poly(ADP-ribose) polymerase cleavage. Additionally, CAERS downregulated the basal expression of anti-apoptotic proteins, including Bcl-2, Bcl-XL, Mcl-1, and survivin, and cell-cycle-related proteins, such as cyclin D1 and c-Myc, while upregulating the pro-apoptotic proteins Noxa and BAD. Collectively, these findings suggest that CAERS triggers apoptosis and enhances the sensitivity of NSCLC cells to cisplatin through a mitochondria-dependent apoptotic pathway (Elkady, 2013).

Fruit fractions of *R. stricta* obtained using n-hexane, chloroform, ethyl acetate, and methanol were evaluated for their cytotoxic, pro-apoptotic, and anti-migratory effects on estrogen receptor–positive (MCF-7), estrogen receptor–negative (MDA-MB-231), and non-tumorigenic (MCF-10A) human breast cell lines (Al-Zharani et al., 2019). All tested extracts demonstrated dose-dependent anti-proliferative effects. Among them, the ethyl acetate fruit extract of *R. stricta* (RSF EtOAc) showed the strongest activity, with an IC_50_ value of 27 μg/mL against MDA-MB-231 cells. However, these fractions also exhibited cytotoxicity toward normal MCF-10A cells, indicating a lack of selectivity between malignant and non-malignant cells. The suppression of cell proliferation by *R. stricta* fractions was linked to the induction of apoptosis, as treated MCF-7 and MDA-MB-231 cells displayed hallmark apoptotic characteristics, including reduced viability, cellular shrinkage, loss of adhesion, and chromatin condensation (Al-Zharani et al., 2019).

Several alkaloids derived from *R. stricta* have been reported to exhibit strong anticancer activity, particularly against breast cancer cell lines. Rhazyaminine was found to significantly decrease the viability of MCF-7 breast cancer cells to nearly 50% and effectively suppress cell migration, as demonstrated by the scratch wound assay. In addition, treatment with rhazyaminine resulted in the reduced expression of multiple genes involved in apoptotic regulation, cell survival, epithelial–mesenchymal transition (EMT), cancer stem cell maintenance, and Wnt signaling pathways in MCF-7 cells (Iqbal et al., 2019).

Razyzmide, a monoterpene indole alkaloid isolated from the aerial parts of *R. stricta*, showed pronounced antiproliferative effects against several cancer cell lines, including MCF-7, HepG2, and HeLa, with IC_50_ values of 5.1 µM. Moreover, exposure to razyzmide led to a marked increase in apoptotic cell populations, reaching 31.4% in MCF-7 cells, 29.2% in HepG2 cells, and 34.9% in HeLa cells (Abdul-Hameed et al., 2022).

Furthermore, studies revealed that treating MCF-7 breast cancer cells with sublethal concentrations of isopicrinine alkaloid extracted from *R. stricta* leaf caused significant changes in gene expression linked to the p53 signaling pathway. Among these, the pro-apoptotic gene *PUMA*, a member of the Bcl-2 family involved in both p53-dependent and independent apoptotic mechanisms, was notably upregulated. At the same time, surviving an anti-apoptotic factor was downregulated. The observed suppression of genes associated with cell division and proliferation further underscores the potential of isopicrinine as a promising anticancer compound (Hajrah et al., 2020).

Recently, Alghamdi et al. (2025) reported the green synthesis of silver nanoparticles (AgNPs) using *R. stricta* extracts, which exhibited pronounced cytotoxic effects against several breast cancer cell lines while showing minimal toxicity toward normal cells. Notably, the *R. stricta*-mediated AgNPs demonstrated the highest anticancer activity against the MDA-MB-231 breast cancer cell line.

### 3.5 Effect of different *R. stricta* leaf and stem extracts on the hemostatic parameters of human plasma

**FIGURE 18:**
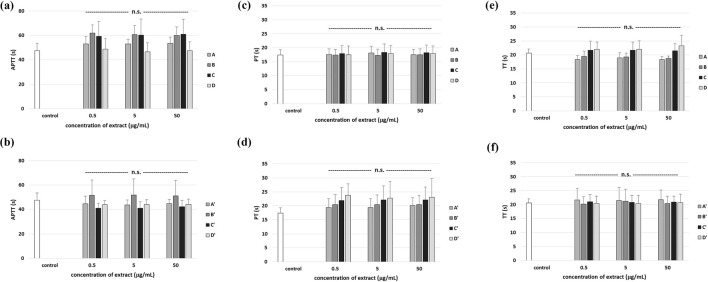
Effects of the four *R. stricta* stem extracts (A–D, concentration range 0.5–50 μg/mL, incubation time: 30 min) and leaf extracts (A′–D′, concentration range 0.5–50 μg/mL, incubation time: 30 min) on the hemostatic parameters (APTT **(a,b)**, PT **(c,d)**, and TT **(f,g)**) of human plasma. Data were expressed as the mean ± SD (n = 6). One-way ANOVA, followed by multicomparison Duncan’s test: n.s, *p* > 0.05 compared with the control (plasma not treated with plant extract).

The coagulation cascade is a complex process that may be modulated by various plant extracts. Phytochemicals have sometimes pro-coagulant and anticoagulant activity. The evaluation of clotting times (PT, TT, and APPT) is a simple and fast screening test, which may study the effect of plant extracts on the coagulation process. In the present study, the effect of extracts from *R. stricta* leaf and stem on these three coagulation times was measured in human plasma *in vitro*. However, our test did not include the influence of leaf and stem extracts on blood clotting times (*in vitro*). Figure 18 shows that none of the tested leaf and stem extracts change coagulation times (APTT, PT, and TT). At the first time, all the obtained results indicate that the tested *R. stricta* extracts did not influence coagulation in human plasma *in vitro*; these results may be interpreted as evidence of hemostatic safety under the tested conditions. Recently, Liu et al. (2025) have found that isorhynchophylline (a tetracyclic oxindole alkaloid, a major component of *Uncaria rhynchophylla*, known for its potential to treat cardiovascular and central nervous system diseases such as hypertension, arrhythmias, amnesia, and dementia) impairs blood platelet hemostatic function without affecting coagulation in mice.

In addition, in the context of hemostasis, oxidative stress plays a regulatory role in platelet activation, endothelial function, and coagulation balance. Indole alkaloids, some of which exhibit antiplatelet or vascular modulating properties, may influence hemostasis indirectly through their redox and mitochondrial effects. As broad pharmacological surveys show, numerous plant-derived indole alkaloids exhibit antioxidant, anti-inflammatory, and antiplatelet activities, suggesting mechanistic links between their redox behavior, membrane protective properties, and vascular homeostasis. By stabilizing cellular redox states and supporting mitochondrial function, indole alkaloids may help preserve endothelial integrity, modulate platelet activity, and influence redox-dependent coagulation pathways, providing a mechanistic basis for contextualizing hemostasis-related findings within their broader biological profile (Omar et al., 2021).

Based on our results, we document for the first time that indole alkaloid metabolites presented in *R. stricta* leaf and stem have antioxidant potential *in vitro*. Table 4 demonstrates that two tested leaf extracts (C′ and D′, at the concentration 5 μg/mL) have an antioxidant potential higher than the other plant extracts used (A–D, A′, and B′). Moreover, preparation C′ does not induce cytotoxicity. The antioxidant potential of extract C′ may be associated with rhazisidine, secamine, and their derivatives.

**TABLE 4:** A comparison of the effects of the four *R. stricta* stem extracts (A-D, concentration 5 µg/mL) and the four *R. stricta* leaf extracts (A'-D', concentration 5 µg/mL) on oxidative damage, coagulation time, and viability of PBMCs. Data represent mean ± SD. Inhibition of oxidative damage by plant preparations is expressed as the percentage of that recorded for control samples (without the plant extract).

	Plant extract							
	A	B	C	D	A’	B’	C’	D’
Oxidative damages								
Inhibition of DNA damage induced by H_2_O_2_ (%)	No effect	No effect	No effect	No effect	No effect	43.7 ± 14.1 (*p* < 0.01)	46.4 ± 9.9 (*p* < 0.01)	61.4 ± 12.1 (*p* < 0.01)
Inhibition of lipid peroxidation induced by H_2_O_2_/Fe^2+^ (%)	No effect	No effect	No effect	No effect	No effect	No effect	41.4 ± 10.4 (*p* < 0.05)	44.5 ± 12.5 (*p* < 0.05)
Inhibition of protein carbonylation induced by H_2_O_2_/Fe^2+^ (%)	No effect	31.4 ± 9.8 (*p* < 0.05)	30.4 ± 11.2 (*p* < 0.05)	No effect	33.3 ± 8.9 (*p* < 0.05)	31.9 ± 9.9 (*p* < 0.05)	25.8 ± 7.8 (*p* < 0.05)	26.7 ± 8.8 (*p* < 0.05)
Coagulation time								
APTT	No effect	No effect	No effect	No effect	No effect	No effect	No effect	No effect
PT	No effect	No effect	No effect	No effect	No effect	No effect	No effect	No effect
TT	No effect	No effect	No effect	No effect	No effect	No effect	No effect	No effect
Viability of PBMCs	No effect	Decrease	Decrease	Decrease	No effect	No effect	No effect	Decrease

Data represent mean ± SD. Inhibition of oxidative damage by plant preparations is expressed as the percentage of that recorded for control samples (without the plant extract).

## 4 Conclusion

The ESI–UPLC–Q-TOF technique is a rapid, sensitive, and reliable method for the tentative identification of active metabolites from *Rhazya stricta*. Fragmentation patterns led to the identification of 10 new indole alkaloids from the leaves and stems, of which 6 are considered important sources for the rapid and accurate detection of *R. stricta* alkaloids. The results obtained indicate that UPLC–Q-TOF is a sensitive and perfectly alternative qualitative method for identifying and detecting plant secondary metabolites. The accurately measured mass values minimized the ambiguity of spectral interpretation by predicting profile patterns of unknown metabolites. Therefore, we propose that the UPLC–Q-TOF technique be developed to produce a database for the sensitive and reliable detection of active metabolomics, including indole alkaloids in *R. stricta* and other natural resources.

Our study documents the promising potential of *R. stricta* leaf and stem extracts containing different indole alkaloid compounds, which can significantly modify the oxidative stress of various elements of blood *in vitro* at relatively low concentrations. In particular, extracts obtained from *R. stricta* leaf exhibit strong antioxidant properties and may have a protective effect under conditions of oxidative stress. However, the tested extracts may contain other metabolites which may affect their antioxidant effects.

The analyses and results we present do have several limitations. It should be emphasized above all that these are *in vitro* studies performed on biological material from many different donors. This can certainly affect the reproducibility of results and the precise determination of the biological activity of *R. stricta* extracts. Furthermore, the chemical and functional diversity of plant compounds, including alkaloids, complicates efforts to generalize their antioxidant activity. This heterogeneity is further amplified by variations in extraction methods, plant sources, solvent polarity, and plant part selection, all of which influence alkaloid yield, purity, and measured antioxidant activity *in vitro*.

A second key limitation arises from the dual antioxidant/pro-oxidant nature of alkaloids, a phenomenon also observed across other phytochemicals. Although many alkaloids demonstrate redox-modulating properties, several studies document their capacity to induce cytotoxicity and oxidative stress under elevated exposures, reflecting their potent bioactivity and narrow therapeutic windows. The challenge of distinguishing therapeutics from toxic doses is further complicated by limited *in vivo* pharmacokinetic and bioavailability data for many alkaloid subclasses.

A third limitation concerns the insufficient mechanistic clarity surrounding the antioxidant actions of alkaloids. While multiple assays (e.g., DPPH, FRAP, hydroxyl radical scavenging) have confirmed antioxidant potential, these biochemical assays alone cannot fully explain cellular or organismal outcomes, nor do they account for metabolic transformation, distribution, or interaction with endogenous redox systems. As a result, the relevance of *in vitro* assay-derived antioxidant capacity to physiological or clinical settings remains uncertain.

The integration of antioxidant measurements with mechanistic studies, such as evaluating redox-sensitive cell signaling, mitochondrial function, or apoptotic pathways, will provide deeper insight into how alkaloids modulate oxidative status within living systems. Further *in vivo* studies, including metabolic fate, pharmacokinetics, and bioavailability, are needed to provide a better understanding of the antioxidant potential of the bioactive metabolites from *R. stricta* and to determine their effectiveness.

## Data Availability

The raw data supporting the conclusions of this article will be made available by the authors without undue reservation.
