# Supplementary material for: Comparative phytochemical, antioxidant, and hemostatic studies of leaf and stem extracts of Rhazya stricta Decne. in human plasma and human peripheral blood mononuclear cells in vitro
Source: Front Mol Biosci. 2026 May 11;13:1786802. doi: 10.3389/fmolb.2026.1786802 (PMC13199043; doi:10.3389/fmolb.2026.1786802)
Supplement: Supplementary file 1 [file Supplementaryfile1.docx]

**Comparative phytochemical, antioxidant, and haemostatic studies of extracts from leaves and stems of *Rhazya stricta* Decne. in human plasma and human peripheral blood mononuclear cells *in vitro***

**Arafa I. Hamed^1^,** **Amal A. A. Mohamed^1^, Mohamed Ali Ben Aissa^2^, Bogdan Kontek^3^, Magdalena Kluska^4^, Katarzyna Woźniak^4^, Mariusz Kowalczyk^5^, Iwona Kowalska^5^, Wiesław Oleszek^6^, Beata Olas^3*^**

*^1^ Phytochemistry Laboratory, Department of Botany, Faculty of Science, Aswan University, Aswan 81528, Egypt,*

*^2^ Department of Chemistry, College of Science, Qassim University, Buraydah 51452, Saudi Arabia,*

*^3^ University of Lodz, Faculty of Biology and Environmental Protection, Department of General Biochemistry, Łódź, Poland,*

*^4^ University of Lodz, Faculty of Biology and Environmental Protection, Department of Molecular Genetics, Łódź, Poland,*

*^5^* *Department of Phytochemistry, Institute of Soil Science and Plant Cultivation, State Research Institute, 24-100 Puławy, Poland*

*^6^* *Centre for Medicinal Plant Cultivation, Institute of Soil Science and Plant Cultivation, State Research Institute, 24-100 Puławy, Poland,*

*****Corresponding authors:** beata.olas@biol.uni.lodz.pl

***Supplementary data (1)***

***High-resolution LC-MS analyses of plant extracts***

High-resolution LC-MS analyses of plant extracts were achieved according to our previous methods (41, 42). Qualitative analyses of indole alkaloids were achieved using high-resolution LC-MS analyses that were linked with a Thermo Scientific Ultimate 3000RS chromatographic system hyphenated to a Bruker Impact II HD (Bruker, Billerica, USA) quadrupole-time of flight (Q-TOF) mass spectrometer. Chromatographic separations were achieved on a Waters BEH C18 column (2.1 × 150 mm, 1.7 µm, Milford, USA). The mobile phase A was formic acid (0.1% (v/v)), and the mobile phase B was acetonitrile mixed with 0.1% (v/v) of formic acid. A concave-shaped gradient (Dionex gradient curve nr 6) from 5% to 80% of phase B over 27 min was applied for separation. The flow rate was 0.5 mL/min, and the column was maintained at 55 °C. Between each injection, the column was equilibrated with 10 column volumes of 5% phase B. The injection volume was 5 µL.

Analyses were performed in positive ion mode [M+H]+ using electrospray ionization. A flow splitter was used to divert the column effluent in a 1:3 ratio between the Q-TOF MS and the charged aerosol detector, which were linked in parallel. CAD gain frequency was 10 Hz. Analyses in the positive ion mode employed recording the UV absorbance trace in the 200-600 nm wavelength range with a 5 nm bandwidth and a 10 Hz acquisition frequency.

Linear (centroid) spectra were gained over a mass range from *m/z* 50 to *m/z* 2000 with the following parameters of the mass spectrometer: positive ion capillary voltage 4.5 kV; dry gas flow 6 l/min; dry gas temperature 200 °C; nebulizer pressure 0.7 bar; collision RF 700.0 V; transfer time 90 μs; pre-pulse storage 7.0 μs. Two precursor ions with intensities over 2000 counts were cracked in each scan. The collision energy and the ion separation width were set automatically, depending on the ion m/z, in the ranges 2.5-35 eV and 2-6 mass units, respectively. The obtained data were internally calibrated using 10 mM sodium formate introduced into the ion source via a 20 µL loop at the beginning of each separation. Processing of the spectra was performed using Bruker Data Analysis 4.3 software.
